# TSG‐6 Protects Against Cerebral Ischemia–Reperfusion Injury via Upregulating Hsp70‐1B in Astrocytes

**DOI:** 10.1111/cns.70354

**Published:** 2025-03-25

**Authors:** Yewei Qu, Lian Yi, Yushi Tang, Fan Yang, Byron Fei Pan, Shanshan Shi, Changda Qu, Fangqin Li, Shirong Wen, Yujun Pan

**Affiliations:** ^1^ Department of Neurology First Affiliated Hospital of Harbin Medical University Harbin Heilongjiang China; ^2^ NHC Key Laboratory of Cell Transplantation First Affiliated Hospital of Harbin Medical University Harbin Heilongjiang China

**Keywords:** apoptosis, astrocyte, cerebral ischemia–reperfusion injury, Hsp70‐1B, TSG‐6

## Abstract

**Aims:**

This study aimed to investigate the relationship between tumor necrosis factor alpha‐induced protein (TNFAIP6/TSG‐6) and astrocytes in cerebral ischemia/reperfusion (I/R) injury.

**Methods:**

Utilizing in vivo and in vitro cerebral I/R models, cerebral infarct volumes, neurobehavioral outcomes, blood–brain barrier (BBB) permeability, as well as indicators of astrocyte apoptosis, reactivity, and A1 phenotype were assessed to evaluate the effects of recombinant rattus TSG‐6 (rrTSG‐6) on astrocytes in acute cerebral I/R injury. Following mRNA sequencing of all astrocyte groups, astrocyte apoptosis and reactivity were analyzed through a combined intervention of rrTSG‐6 and Apoptozole, a heat shock protein 70‐1B (Hsp70‐1B) inhibitor, in vitro.

**Results:**

The findings demonstrated that rrTSG‐6 significantly reduced cerebral infarct volumes by nearly half, improved neurobehavioral outcomes, mitigated BBB damage, and suppressed the expressions of astrocyte apoptosis markers, reactivity indicators, and A1 phenotype markers. mRNA sequencing revealed that the Hsp70‐1B protein level increased to approximately 1.6 times that of the rrTSG‐6 non‐intervention group. Furthermore, Apoptozole impeded the expressions of astrocyte apoptosis markers, reactivity indicators, and A1 phenotype markers.

**Conclusion:**

TSG‐6 inhibited nuclear factor kappa‐B (NF‐κB) phosphorylation by upregulating Hsp70‐1B in oxygen–glucose deprivation/reoxygenation (OGD/R)‐induced astrocytes, thereby exerting a protective effect through anti‐apoptotic mechanisms and the suppression of astrocyte reactivity and A1 transformation following cerebral I/R injury.

## Graphical abstract

TSG‐6 diminishes infarct volume, enhances neurological function, and mitigates blood–brain barrier damage following acute cerebral ischemia–reperfusion injury. Furthermore, TSG‐6 exerts neuroprotective effects by upregulating Hsp70‐1B to inhibit NF‐κB phosphorylation in oxygen–glucose deprivation/reoxygenation‐induced astrocytes, thereby suppressing apoptosis, astrocyte reactivity, and A1 phenotype transformation.

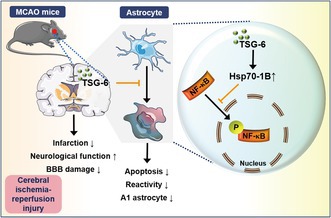

**Abbreviations**
ANOVA: analysis of variance
Az: apoptozole
BBB: blood–brain barrier
BP: biological process
C3: complement 3
CC: cellular component
CCA: common carotid artery
cDNA: complementary deoxyribonucleic acid
CNS: central nervous system
DDIT3/CHOP: DNA‐damage‐inducible transcript 3
DEGs: differential expressed genes
ECA: external carotid artery
ER: endoplasmic reticulum
ERS: endoplasmic reticulum stress
FBS: fetal bovine serum
FC: fold change
FITC: fluorescein isothiocyanate
FPKM: fragments per kilobase of exon model per million mapped fragments
GAPDH: glyceraldehyde‐3‐phosphate dehydrogenase
GEO: Gene Expression Omnibus
GFAP: glial fibrillary acid protein
GO: Gene Ontology
GRP78: glucose‐regulated protein 78
Hsp: heat shock protein
I/R: ischemia–reperfusion
ICA: internal carotid artery
ICH: intracerebral hemorrhage
IF: immunofluorescence
IL: interleukin
INOS: inducible nitric oxide synthase
IS: ischemic stroke
JAK2: Janus Kinase 2
KEGG: Kyoto Encyclopedia of Genes and Genomes
LPS: lipopolysaccharide
MCAO/R: middle cerebral artery occlusion/reperfusion
MF: molecular function
MMP9: matrix metalloproteinases 9
mNSS: modified neurological severity score
mRNA: messenger ribonucleic acid
mRNA‐seq: messenger ribonucleic acid sequencing
MSCs: mesenchymal stem cells
OGD/R: oxygen–glucose deprivation/reperfusion
PBS: phosphate buffer saline
pNF‐κB: phosphorylated nuclear factor kappa B
RrTSG‐6: recombinant rattus tumor necrosis factor alpha induced protein 6
RT‐qPCR: reverse transcription quantitative real‐time polymerase chain reaction
S100A10: S100 calcium binding protein A10
SAH: subarachnoid hemorrhage
SCI: spinal cord injury
SD: Sprague–Dawley
sd: standard deviation
STAT3: signal transducer and activator of transcription 3
TNFAIP6/TSG‐6: tumor necrosis factor alpha induced protein 6
TNF‐α: tumor necrosis factor alpha
TTC: 2,3,5‐triphenylterazolium chloride
TUNEL: terminal‐deoxynucleotidyl transferase mediated nick end labeling
WB: western blot
ZO‐1: zonula occludens 1
β‐actin: beta‐actin

## 1 Introduction

Tumor necrosis factor alpha‐induced protein 6 (TNFAIP6), also known as TSG‐6, is a multifunctional protein encoded by the *Tnfaip6* gene, characterized by highly conserved amino acid sequences, and involved in various biological processes [1]. For instance, TSG‐6 expression is upregulated in brain tissue with increasing neuropathological severity in patients with Alzheimer's disease, possibly suggesting a failed tissue‐repair response [2]. Moreover, TSG‐6 exerts protective anti‐inflammatory effects in several neurological disorders, including multiple sclerosis [3], traumatic brain injury [4], intracerebral hemorrhage (ICH) [5], subarachnoid hemorrhage (SAH) [6, 7], ischemic stroke (IS) [8, 9, 10, 11, 12, 13], and periventricular leukomalacia [14]. The pathophysiology of IS is complex, involving excitotoxicity, oxidative stress, reperfusion injury, inflammation, among other factors [15]. A previous study on human brain tissues with acute IS demonstrated that TSG‐6 expression was upregulated in most stroke‐affected regions, including both peri‐infarcted and infarcted regions, while its expression remained low in unaffected tissue [8]. This finding is subsequently corroborated in models of global cerebral ischemia–reperfusion (I/R) injury and middle cerebral artery occlusion/reperfusion (MCAO/R) [9, 10, 11, 12]. Furthermore, TSG‐6 is shown to enhance neurological function [9, 10, 11, 12, 13], reduce neutrophil elastase and pro‐inflammatory cytokines [9], promote the expression of M2 microglial markers in ischemic brain regions [10], and mitigate neuronal damage by suppressing endoplasmic reticulum stress‐mediated inflammation [11]. A recent study indicated that ischemic preconditioning conferred neuroprotection following acute IS by further inducing endogenous TSG‐6 expression [12]. Based on previous studies, endogenous TSG‐6 levels increased in both brain tissue and peripheral blood following acute IS; however, the correlation between these two compartments warrants further investigation [9, 10, 11, 12, 13, 16].

Astrocytes play a crucial role in the development and maintenance of a healthy central nervous system (CNS), fulfilling multiple essential functions in acute and chronic CNS disorders, including providing structural and metabolic support, preserving blood–brain barrier (BBB) integrity, mediating neuroinflammation, regulating oxidative stress, and promoting synaptic formation and maintenance [17]. With advancements in research methodologies, increasing attention has been directed toward the role of astrocytes in CNS diseases [18]. Astrocytes exhibit diverse phenotypes across different temporal and spatial contexts, and modulating astrocyte reactivity presents new opportunities and potential research targets [19]. Under inflammatory conditions, A1 astrocytes, which express high levels of neuroinflammatory genes, predominate in the early stages, whereas A2 astrocytes, which express neuroprotective genes, are more prevalent during the initial phase of IS [20]. Notably, these phenotypes coexist in various diseases but differ in proportional representation [19, 20]. During IS, astrocyte activation is characterized by the upregulated expression of glial fibrillary acidic protein (GFAP), which is widespread and persistent [21]. Due to the heterogeneity of reactive astrocytes, certain pro‐inflammatory astrocytes compromise BBB integrity by increasing the expression of pro‐inflammatory factors such as chemokines, cytokines, inducible nitric oxide synthase (iNOS), and matrix metalloproteinase (MMP) [21]. Conversely, anti‐inflammatory astrocytes facilitate BBB repair by resolving inflammation [21]. Currently, limited research has been conducted on the regulatory mechanisms of TSG‐6 in astrocytes. The first study to address this topic, published in 2012, reported that the conditioned medium from lipopolysaccharide (LPS)‐stimulated astrocytes induced TSG‐6 expression in human placenta‐derived mesenchymal stem cells (MSCs); however, no further mechanistic investigations were conducted [3]. Subsequent studies have suggested that TSG‐6 may exert an anti‐inflammatory effect within the glial scar [22, 23]. Additionally, research has demonstrated that bone marrow‐derived MSCs contribute to BBB repair and reduce astrocyte apoptosis by secreting TSG‐6, which inhibits the nuclear factor kappa‐B (NF‐κB) signaling pathway [5, 7]. Furthermore, TSG‐6 has been shown to mitigate early brain injury following SAH by suppressing CARD domain containing 4 inflammasome‐mediated astrocyte pyroptosis [24].

In view of no relevant mechanism studies between the two in acute IS, we used recombinant rattus TSG‐6 (rrTSG‐6) to intervene in both the MCAO/R model of rats in vivo and the oxygen–glucose deprivation/reperfusion (OGD/R) model of astrocytes in vitro. Messenger ribonucleic acid sequencing (mRNA‐seq) of astrocytes in vitro intervention was performed to investigate the regulatory mechanism of TSG‐6 on astrocytes in acute cerebral I/R injury. Our research aimed to further investigate the effect of TSG‐6 on astrocyte responses and phenotype regulation, in order to provide a little foundation for IS treatment by using TSG‐6 as an intervention target or a clinical transformation of exogenous application.

## 2 Materials and Methods

### 2.1 Animals

SPF male Sprague–Dawley (SD) rats and pregnant rats were purchased from the Faculty of Laboratory Animal of Harbin Medical University. All animal experiments were approved by the Institutional Animal Care and Use Committee of the First Affiliated Hospital of Harbin Medical University (IACUC application: 2019050) and were conducted in accordance with the guidelines of the National Institutes of Health for the care and use of laboratory animals. The animals were housed in the Experimental Animal Center of the First Affiliated Hospital of Harbin Medical University under a 12‐h light/dark cycle at 24°C, with food pellets and water available ad libitum. A total of 114 healthy male SD rats were utilized, 26 of which were excluded (see Experimental Design below). In experiment 1, rats were randomly assigned to four groups: the sham operation group, MCAO for 2 h followed by reperfusion for 12 h, MCAO for 2 h followed by reperfusion for 24 h, and MCAO for 2 h followed by reperfusion for 72 h. Six rats were excluded due to various reasons (three due to subarachnoid hemorrhage: two from the MCAO‐2 h/R‐24 h group and one from the MCAO‐2 h/R‐48 h group; one mortality post‐surgery in the MCAO‐2 h/R‐72 h group; and two unsuccessful MCAO/R model inductions: one from the MCAO‐2 h/R‐48 h group and one from the MCAO‐2 h/R‐72 h group). In experiment 2, rats were randomly assigned to three groups using a random number table: the sham operation group (sham group), MCAO/R with phosphate‐buffered saline (PBS) treatment group (MCAO/*R* + PBS group), and MCAO/R with rrTSG‐6 treatment group (MCAO/*R* + rrTSG‐6 group). Twenty rats were excluded for various reasons (eight due to subarachnoid hemorrhage: four from the MCAO + PBS group and four from the MCAO + rrTSG‐6 group; eight mortalities post‐surgery: five from the MCAO + PBS group and three from the MCAO + rrTSG‐6 group; one mortality post‐anesthesia in the MCAO/*R* + PBS group; three unsuccessful MCAO/R model inductions: one from the MCAO + PBS group and two from the MCAO/*R* + rrTSG‐6 group).

### 2.2 Experimental MCAO/R Model

The MCAO/R model was established as previously described [25]. Rats weighing 240–260 g were anesthetized by intraperitoneal injection of 3% phenobarbital sodium (40 mg/kg). At the central entry of the neck, muscles and fascia were carefully dissected to expose the common carotid artery (CCA), external carotid artery (ECA), and internal carotid artery (ICA). The thread (Jialing Biotechnology Co. Ltd., Guangzhou, China) was gradually inserted into the distal end of the ECA after excision. The filament was secured upon encountering slight resistance, indicating occlusion of the origin of the middle cerebral artery. Following 2 h of ischemia, the filament was withdrawn to allow reperfusion, and the skin was sutured after disinfection. Throughout the ischemic period, anesthesia was maintained, and eyelid and pedal reflexes were routinely assessed to ensure adequate anesthesia. Post‐awakening, the rats were placed on a warm pad and maintained in an appropriate environment with sufficient food and water. Sham‐operated animals underwent identical procedures, except for arterial occlusion. The MCAO/R model was deemed successful based on the Zea‐Longa suture‐occluded criteria [26]. Neurological deficits were scored as follows: 0, no neurological impairment; 1, inability to fully contract the contralateral forelimb upon tail elevation; 2, spontaneous circling behavior; 3, severe postural leaning; 4, absence of spontaneous locomotion and loss of consciousness [26]. Rats scoring 2–3 were considered successfully modeled and included in further experiments.

### 2.3 Intra‐Arterial Drug Administration via the ICA

RrTSG‐6 protein was synthesized by Boaoxin Biotechnology Co. Ltd. (Guangzhou, China) (Figure S1). RrTSG‐6 was dissolved in sterile PBS (Hyclone, USA) at a final concentration of 0.05 μg/μl. An injection device comprising a 31G insulin needle and a polypropylene capillary transparent hose was employed to administer 100 μL PBS or rrTSG‐6 (5 μg) [6] following filament removal (Figure S2). The injection device was introduced through the residual ECA incision (thread insertion site) and advanced along the ICA to the pterygopalatine artery branch. The procedure was performed in accordance with previous studies [27, 28].

### 2.4 Primary Astrocyte Culture and Identification

Neonatal SD rats within 24 h of birth were obtained from the Faculty of Laboratory Animal of Harbin Medical University. Upon arrival at the laboratory, neonatal SD rats were subjected to hypothermia anesthesia for 1 min, followed by immersion in a 75% alcohol beaker for whole‐body disinfection for approximately 1 min. Brain tissues were subsequently isolated and immediately transferred to pre‐cooled D‐Hank's balanced salt solution (Hyclone, USA). The cerebral cortex was dissected, minced into ~1 mm^3^ fragments, and incubated with 0.25% trypsin at 37°C. The suspension was repeatedly pipetted to obtain a single‐cell suspension, which was subsequently filtered and centrifuged at 1000 rpms for 10 min. The cell pellet was resuspended in a cell culture medium (DMEM/F12, Hyclone, USA) and cultured in a T75 flask containing 15 mL of medium supplemented with 10% fetal bovine serum(FBS) (Gibco, USA) and 1% antibiotic solution (EpiZyme Ltd., Shanghai, China) at 37°C in a 5% CO_2_ incubator for 1 h. The cell suspension was then transferred to a new T75 flask for continued culture. After 7–9 days, the flasks underwent two horizontal oscillations to detach cells, which were subsequently subcultured and cryopreserved in liquid nitrogen for future use [29].

Primary astrocytes at passage 1 (P1) were selected for further investigation and characterization. Briefly, after being washed three times with PBS, the astrocytes were fixed with 4% paraformaldehyde for 15 min. Subsequently, the cells were permeabilized using 0.3% Triton X‐100 for 15 min and washed with PBS. Astrocyte‐specific markers, including anti‐GFAP (1:50, ab279290, Abcam, UK), were applied overnight at 4°C. On the following day, the cells were incubated with a secondary antibody (Cy3‐conjugated AffiniPure goat anti‐mouse IgG, 1:200, SA00009‐1, ProteinTech Group, USA) at 37°C for 1 h, followed by nuclear staining with DAPI (Beyotime, P0131, Shanghai, China) for 15 min. The stained cells were subsequently examined under a fluorescence microscope (Zeiss, Germany). Primary astrocytes were considered successfully identified when their purity exceeded 95%.

### 2.5 Cell Modeling and Grouping

Prior to experimental treatment, primary astrocytes were washed three times with PBS. The medium was then replaced with glucose‐free Earle's balanced salt solution (EBSS) (Biotopped Ltd., Beijing, China) and incubated for 6 h under humid conditions with a gas mixture of 1% O_2_, 5% CO_2_, and 94% N_2_ at 37°C. Subsequently, the medium was replaced by complete culture media (DMEM/F12 with 10% FBS and 1% penicillin and streptomycin) under normoxic conditions for 24 h.

In the first in vitro experiment, astrocytes were allocated into three groups: (1) the control group, in which cells were maintained in complete medium under normoxic conditions; (2) the OGD/*R* + PBS group, in which cells were subjected to OGD for 6 h followed by normal culture conditions with PBS treatment for 24 h; and (3) the OGD/*R* + rrTSG‐6 group, in which cells were exposed to OGD followed by normal culture conditions with rrTSG‐6 treatment at a predetermined screening concentration for 24 h. In the second in vitro experiment, astrocytes were divided into the four groups: (1) control, (2) OGD/*R* + PBS, (3) OGD/*R* + rrTSG‐6, and (4) OGD/*R* + rrTSG‐6 combined with Apoptozole (Az) (HY‐15098, MedChemExpress, USA) at the screening concentration.

### 2.6 Neurological Function Assessment

Behavioral recovery was evaluated by an independent examiner blinded to experimental design, using the modified Neurological Severity Score (mNSS) [30] and adhesive‐removal test [31] on days 1, 3, and 7 following MCAO surgery. The mNSS ranges from 0 to 18 scores, with a score of 0 indicating normal function and 18 representing maximal deficit. A single point represented an inability to perform a test or lack of a tested reflex. To administer the adhesive‐removal test, a round paper sticker with an area of 113.1 mm^2^ was attached to the underside of the front paws of each animal. The animals were trained 3 days before surgery. The adhesive‐removal time of the affected side (Left) was measured before and after theoperation.

### 2.7 2,3,5‐Triphenyl Tetrazolium Chloride (TTC) Staining

TTC staining was performed to assess cerebral infarction size. At 24 h post‐MCAO/R, rats were euthanized using a lethal dose of phenobarbital (100 mg/kg). Brain tissues were immediately excised and frozen at −20°C for 20 min. Coronal sections of 2 mm thickness were then prepared and incubated in a 2% TTC dye solution (Solarbio Science & Technology Co. Ltd., Beijing, China) at room temperature in the dark for approximately 20 min. The sections were subsequently immersed in a 4% paraformaldehyde solution for 2 h. Normal brain tissue was stained red, whereas infarcted areas appeared pale gray. Infarct volume was quantified using ImageJ software according to the formula: Percentage of cerebral infarct volume = (sum of infarct volumes of all sections)/(sum of total section volumes) × 100%.

### 2.8 Western Blot (WB) Analysis

WB analysis was employed to evaluate protein expression associated with BBB damage, astrocyte reactivity, and apoptosis. Protein samples were extracted from ischemic hemispheric brain tissues or astrocyte cell pellets using RIPA lysis buffer (Biosharp, Anhui, China) supplemented with phosphatase and protease inhibitors (Roche, Germany). A total of 30 μg of protein was separated via SDS‐PAGE and transferred onto polyvinylidene difluoride membranes (Millipore, Bedford, MA, USA). Membranes were incubated at 4°C overnight with primary antibodies. The primary antibodies were as follows: rabbit polyclonal anti‐TSG‐6 antibody (1:500, AF5492, Affinity, USA), rabbit polyclonal anti‐zonula occludens 1 (ZO‐1) antibody (1:500, 21773‐1‐AP, ProteinTech Group, USA), rabbit polyclonal anti‐occludin antibody (1:1000, ab216327, Abcam, UK), rabbit polyclonal anti‐MMP9 antibody (1:500, 10375‐2‐AP, ProteinTech Group, USA), rabbit polyclonal anti‐iNOS antibody (1:500, YT3169, Immunoway, USA), rabbit polyclonal anti‐cleaved‐caspase3 antibody (1:500, YC0006, Immunoway, USA), mouse monoclonal anti‐GFAP antibody (1:1000, ab279290, Abcam, UK), rabbit polyclonal anti‐complement 3 (C3) antibody (1:2000, ab200999, Abcam, UK), mouse polyclonal anti‐heat shock protein 70 (Hsp 70) antibody (1:1000, ab5439, Abcam, UK), rabbit polyclonal anti‐glucose regulated protein 78 (GRP78) antibody (1:300, AF5366, Affinity, USA), rabbit polyclonal anti‐C/EBP‐homologous protein (CHOP) antibody (1:400, AF5280, Affinity, USA), rabbit polyclonal anti‐NF‐κB antibody (1:500, AF5006, Affinity, USA), rabbit polyclonal anti‐phospho‐NF‐κB (p‐NF‐κB) (Ser536) antibody (1:500, AF2006, Affinity, USA), rabbit polyclonal anti‐glyceraldehyde‐3‐phosphate dehydrogenase (GAPDH) antibody (1:1000, AF7021, Affinity, USA), rabbit polyclonal anti‐beta‐Actin (β‐actin) antibody (1:1000, AF7018, Affinity, USA). Horseradish peroxidase‐conjugated secondary antibodies were applied at a 1:5000 dilution (Goat anti‐mouse, goat anti‐rabbit) (Affinity, USA) for 1 h at room temperature. Protein bands were detected using an enhanced chemiluminescence system, and GAPDH or β‐actin was used as a loading control. Densitometric analysis was performed using ImageJ software.

### 2.9 Terminal Deoxynucleotidyl Transferase dUTP Nick‐End Labeling (TUNEL) Staining

To assess cell death as an indicator for tissue integrity, TUNEL staining was conducted using the TUNEL Apoptosis Assay Kit (T2130, Solarbio Science & Technology Co. Ltd., Beijing, China) in accordance with the manufacturer's instructions. Brain sections (5 μm) were fixed with 4% paraformaldehyde for 15 min and subsequently washed with PBS. The sections were then permeabilized using 0.3% Triton X‐100 for 15 min and blocked with 10% donkey serum (Solarbio Science & Technology Co. Ltd., SL050, Beijing, China) for 1 h. TUNEL working solution was added, and the samples were incubated at 37°C for 60 min in the dark. Following the removal of the TUNEL working solution and PBS washing, the sections were treated with reaction buffer and stained with Hoechst to visualize nuclei. Fluorescence intensity was quantified using a fluorescence microscope equipped with a fluorescein isothiocyanate (FITC) channel (Zeiss, Germany). TUNEL‐positive cells were counted, and the apoptotic index was calculated as a ratio of TUNEL‐positive cells to the total cell count per field.

### 2.10 Immunofluorescence (IF) Staining

Brains sections and astrocyte slides were fixed, permeabilized, and blocked using the same protocol as TUNEL staining. Then, the samples were incubated overnight at 4°C with the following primary antibodies: GFAP (1:50, ab279290, Abcam, UK) and C3 (1:2000, ab200999, Abcam, UK). On the following day, the samples were incubated with secondary antibodies (Cy3‐conjugated AffiniPure goat anti‐mouse IgG and fluorescein‐conjugated AffiniPure goat anti‐rabbit IgG, 1:200, SA00009‐1/SA00003‐2, ProteinTech Group, USA) at 37°C for 1 h. Nuclei were stained using DAPI (Beyotime, P0131, Shanghai, China). Fluorescence images were captured using a fluorescence microscope (Zeiss, Germany), and immunofluorescence intensity was quantified using ImageJ software.

### 2.11 Cell Viability Assay

Cell viability was assessed using the 96 AQueous One Solution Reagent (G3580, Promega, USA) according to the manufacturer's instructions. Briefly, cell density was adjusted to 5 × 10^3^ cells per well in a 96‐well plate containing 100 μL of complete medium. Astrocytes in experimental groups were subjected to OGD for 6 h, followed by normoxic culture with either varying concentrations of rrTSG‐6 (0, 200, 400, 600 ng/mL) or a combination of rrTSG‐6 at the screening concentration and different concentrations of Az (0, 5, 10, 15 μmol/L). Subsequently, 20 μL of 3‐(4,5‐dimethylthiazol‐2‐yl)‐5‐(3‐carboxymethoxyphenyl)‐2‐(4‐sulphophenyl)‐2H‐Tetrazolium (MTS) reagent was added to each well and incubated for 2 h at 37°C in a 5% CO_2_ atmosphere in the dark. Absorbance (optical density—OD) values at 490 nm were measured using a microplate reader to determine the optimal intervention concentration.

### 2.12 Data Processing, Identification of Differentially Expressed Genes (DEGs), and Gene Enrichment Analysis

Normalized GSE35338 expression data were obtained from the Gene Expression Omnibus (GEO) database (http://www.ncbi.nlm.nih.gov/geo/) [32]. The GSE35338 dataset is based on the GPL1261 platform (Affymetrix Mouse Genome 430 2.0 Array). Expression data of five astrocyte samples collected 24 h after MCAO were extracted. Gene symbols were annotated based on the GPL1261 platform, and probes without corresponding gene symbols or mapping to multiple genes were filtered out. The mean expression values were calculated for genes represented by multiple probes. Differential expression analysis was conducted using the limma package in R [33]. Astrocyte samples were categorized into *Tnfaip6*‐high and *Tnfaip6*‐low expression groups, with the median *Tnfaip6* expression level serving as the cutoff. DEGs between these groups were identified using lmFit and eBayes functions in the limma package, with a cutoff threshold set at *p* < 0.05 and an absolute log2‐fold change (|log2 FC|) ≥ 0.5. Gene Ontology (GO) functional enrichment analysis, including biological process (BP), cellular component (CC) and molecular function (MF) categories [34], along with Kyoto Encyclopedia of Genes and Genomes (KEGG) pathway analysis [35], was performed using the Metascape online tool (http://metascape.org/gp/index.html#/main/step1) [36]. A statistical significance threshold of *p* < 0.05 was applied.

### 2.13 mRNA‐Seq

The mRNA‐seq analysis of the nine cell samples, comprising three samples per group (control, OGD/*R* + PBS and OGD/*R* + rrTSG‐6), was conducted to Aqu Biotechnology Co. Ltd. (Shanghai, China). Total RNA was extracted and purified using TRIzol reagent (Invitrogen, USA) following the manufacturer's procedure. The RNA concentration and purity of each sample were quantified using the NanoDrop ND‐1000 (NanoDrop, USA). RNA integrity was assessed using the Bioanalyzer 2100 (Agilent, USA) with a RNA integrity number (RIN) > 7.0 and further confirmed via electrophoresis on a denaturing agarose gel. Poly (A) RNA was purified from 1 μg of total RNA using the Dynabeads Oligo (dT)25‐61005 (Thermo Fisher, USA) through two rounds of purification. The purified poly(A) RNA was then fragmented into small fragments using Magnesium RNA Fragmentation Module (cat.e6150, NEB, USA) under 94°C 5–7 min. The cleaved RNA fragments were reverse‐transcribed to synthesize complementary deoxyribonucleic acid (cDNA) using SuperScript II Reverse Transcriptase (cat.1896649, Invitrogen, USA), followed by the synthesis of U‐labeled second‐strand DNA using *Escherichia coli* DNA polymerase I (cat.m0209, NEB, USA), RNase H (cat.m0297, NEB, USA) and dUTP Solution (cat.R0133, Thermo Fisher, USA). Subsequently, an A‐base was then added to the blunt ends of each strand to facilitate adapter ligation. The adapters, containing a T‐base overhang were ligated to the A‐tailed fragmented DNA. Single‐ or dual‐index adapters were ligated, followed by size selection using AMPureXP beads. The U‐labeled second‐stranded DNA was then treated with a heat‐labile UDG enzyme (cat.m0280, NEB, USA), and the ligated products were amplified using PCR under the following conditions: initial denaturation at 95°C for 3 min; 8 cycles of denaturation at 98°C for 15 s, annealing at 60°C for 15 s, and extension at 72°C for 30 s; followed by a final extension at 72°C for 5 min. The final cDNA library had an average insert size of 300 ± 50 bp. Finally, 2 × 150 bp paired‐end sequencing (PE150) was performed on an Illumina Novaseq 6000 platform (LC‐Bio Technology CO. Ltd., Hangzhou, China) following the manufacturer's instructions.

Quality control of sequencing data was conducted using Cutadapt software (https://cutadapt.readthedocs.io/en/stable/, version: cutadapt‐1.9) to remove reads containing adaptor contamination (command line: ~cutadapt ‐a ADAPT1 ‐A ADAPT2 ‐o out1.fastq ‐p out2.fastq in1.fastq in2.fastq ‐O 5 ‐m 100). Low quality bases and undetermined bases were removed before mapping reads to the genome using HISAT2 (https://daehwankimlab.github.io/hisat2/, version: hisat2‐2.0.4) (command line: ~hisat2 ‐1 R1.fastq.gz ‐2 R1.fastq.gz ‐S sample_mapped.sam). The mapped reads of each sample were assembled using StringTie (http://ccb.jhu.edu/software/stringtie/, version: stringtie‐1.3.4d.Linux_x86_64) with default parameters (command line: ~stringtie ‐p 4 ‐G genome.gtf ‐o output.gtf ‐l sample input.bam). The transcriptomes from all samples were subsequently merged using gffcompare (http://ccb.jhu.edu/software/stringtie/gffcompare.shtml, version: gffcompare‐0.9.8.Linux_x86_64) to construct a comprehensive transcriptome. StringTie and ballgown (http://www.bioconductor.org/packages/release/bioc/html/ballgown.html) were then employed to quantify transcript expression levels using Fragments Per Kilobase of exon model per Million mapped fragments (FPKM) (FPKM = [total_exon_fragments/mapped_reads(millions) × exon_length(kB)]) (command line: ~stringtie ‐e ‐B ‐p 4 ‐G merged.gtf ‐o samples.gtf samples.bam).

The quality control results are as follows. After filtering out unqualified sequences, valid data from nine samples were obtained, with statistics on data volume and base content presented in Table S1. Reads were then mapped to the reference genome, yielding the following read counts per sample: 45,226,510; 39,798,004; 51,080,466; 41,844,658; 39,121,510; 49,867,586; 52,553,840; and 52,110,828. (1) The number of reads mapped to the genome (total mapped) and transcriptome data utilization ratios exceeded 83.5%, with more than 70% of the data being usable for all samples. (2) The number and proportion of reads uniquely mapped to the reference sequences. (3) The number and proportion of reads with multiple mapping positions on the reference sequences. (4) The number and proportion of paired‐end reads mapped to the reference genome (PE mapped). (5) The number and proportion of reads mapped to the sense strand. (6) The number and proportion of reads mapped to the antisense strand. (7) The number and proportion of reads spanning two or more splice junctions, where splice read proportions depended on sequencing fragment length. (8) The number and proportion of non‐spliced reads mapped entirely to exons. The read counts and proportions for all nine samples are summarized in Tables S2 and S3, while additional quality control data are presented in Figure S3.

DEGs were identified using the R package edgeR [33] (http://www.bioconductor.org/packages/release/bioc/HTML/DESeq2.html) with |log2 FC| ≥ 0.5 and adjust (adj.) *p* < 0.05. GO [34] and KEGG [35] enrichment analyses were conducted to explore the biological functions and underlying mechanisms of the DEGs using the Metascape tool (https://metascape.org/gp/index.html#/main/step1) [36] and the KOBAS‐i database (http://kobas.cbi.pku.edu.cn/, version: KOBAS 3.0) [37].

### 2.14 Reverse Transcription Quantitative Real‐Time Polymerase Chain Reaction (RT‐qPCR)

Total RNA was extracted from rat brain tissues and primary astrocytes using TRIzol reagent (Invitrogen, USA). The RNA concentration and purity were assessed using a NanoDrop Microvolume Spectrophotometer, ensuring OD 260/280 values of 1.9–2.1 and OD 260/230 values of 1.9–2.2 for usability. cDNA was synthesized from total RNA using a Reverse Transcription Kit (TOYOBO, Japan). RT‐qPCR was performed in a 10 μL reaction system using 2× SYBR Green qPCR Master Mix II (Universal) (Seven, Beijing, China). All primers were synthesized by General Biol Co. (Anhui, China) and their sequences are listed in Table S4. The RT‐qPCR was conducted using a two‐step method with the following cycling conditions: initial denaturation at 95°C for 30 s, followed by 40 cycles of denaturation at 95°C for 15 s, and annealing/extension at 60°C for 25 s. The relative expression levels of target genes were calculated using the 2^−ΔΔ*CT*^ method.

### 2.15 Flow Cytometry Analysis

Apoptosis was evaluated using the Annexin V‐FITC and propidium iodide (PI) Apoptosis Detection Kit (CA1020, Solarbio Science & Technology Co. Ltd., Beijing, China) according to the manufacturer's instructions. Briefly, following treatment, astrocytes were harvested using EDTA‐free trypsin and washed twice with pre‐chilled PBS. The cells were resuspended in 1× binding buffer and adjusted to a concentration of 2 × 10^6^ cells/mL. A 100 μL aliquot of the suspension was transferred to a flow cytometry tube, mixed with 5 μL of Annexin V‐FITC, and incubated in the dark at room temperature for 5 min. Subsequently, 5 μL of PI and 400 μL of PBS were added, and apoptotic and necrotic cells were quantified using a flow cytometer.

### 2.16 Statistical Analysis

All data were subjected to the Shapiro–Wilk normality test and the homogeneity test of variance. If the data followed a normal distribution and exhibited homogeneity of variance, they were expressed as means ± standard deviation (sd), and one‐ or two‐way analysis of variance (ANOVA) followed by Tukey's post hoc test was applied. If the data did not conform to normal distribution, they were expressed as the median with interquartile range and analyzed using a non‐parametric test (Kruskal–Wallis test). Data following a normal distribution but demonstrating heterogeneous variance were analyzed using the Brown–Forsythe test followed by Games‐Howell post hoc test. For bar graphs, a representative experiment from at least three independent experiments was presented, with each data point representing one independent biological replicate. Statistical analyses were conducted using GraphPad Prism 8.0. Differences were considered statistically significant at *p* < 0.05.

## 3 Results

### 3.1 RrTSG‐6 Alleviates Brain Damage and Astrocyte Apoptosis Following Acute Cerebral I/R Injury

**FIGURE 1:**
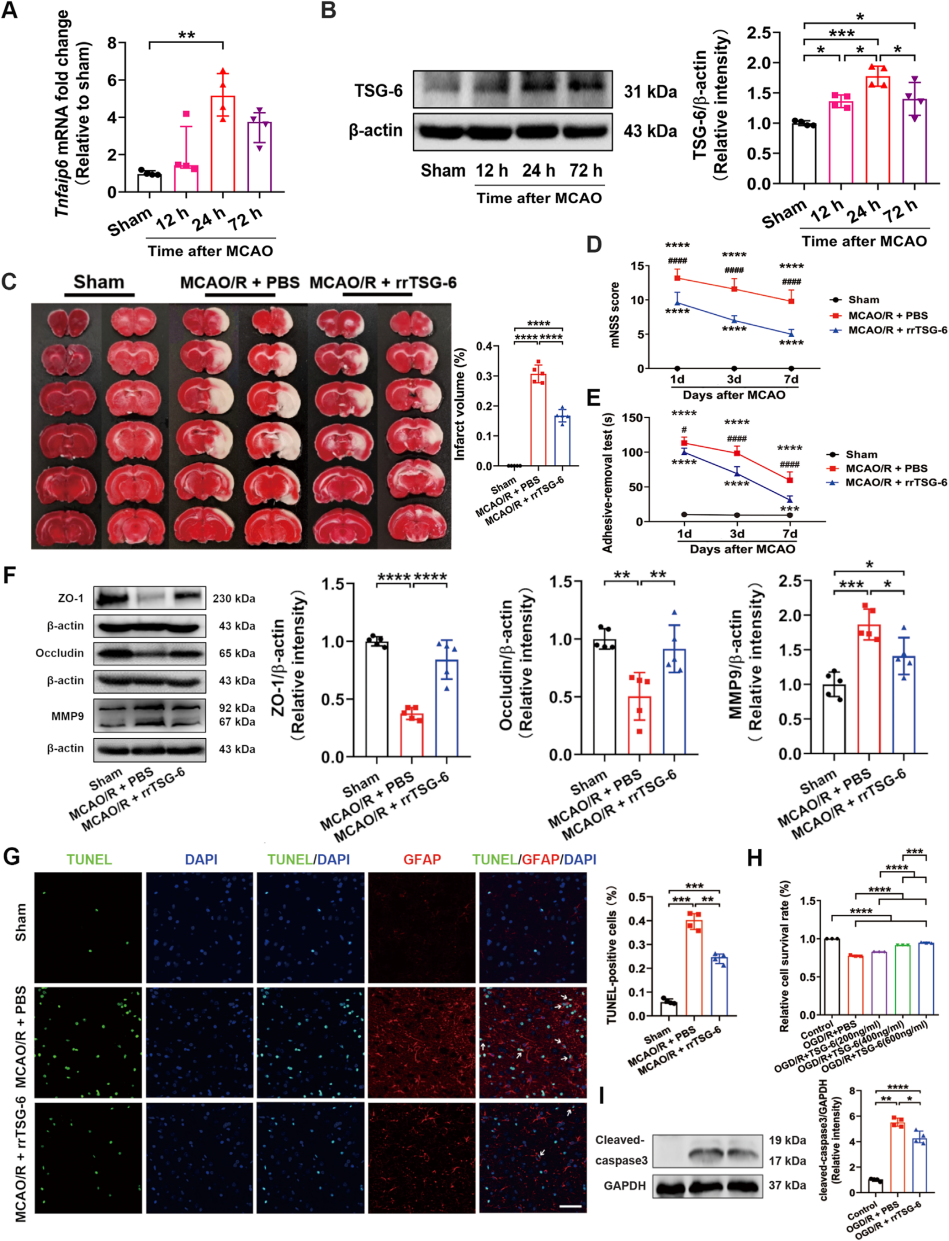
RrTSG‐6 alleviated brain damage and astrocyte apoptosis following acute cerebral I/R injury. (A) mRNA levels of *Tnfaip6* and (B) protein levels of TSG‐6 were detected respectively by RT‐qPCR and WB at 12, 24 and 72 h post‐MCAO/R (*n* = 4); (C) TTC staining and quantitative analysis of lesion volume were shown at 24 h post‐MCAO/R (*n* = 5); (D) mNSS and (E) Adhesive‐removal test used to evaluate the neurological function of rats on the 1st, 3th and 7th days following MCAO/R (*n* = 5); (F) the protein levels of ZO‐1, occluding and MMP9 were detected by WB at 24 h post‐MCAO/R (*n* = 5); (G) At 24 h post‐MCAO/R, representative photomicrographs showed TUNEL+ cells and TUNEL^+^/GFAP^+^ cells in the ischemic hemisphere cortex among sham, MCAO/*R* + PBS and MCAO/*R* + rrTSG‐6 groups, and quantitative data of immunopositive cells for TUNEL were shown (*n* = 4), green represented TUNEL, red represented GFAP, blue represented DAPI and white arrows represented apoptotic astrocytes, bar = 50 μm; (H) At 24 h post‐OGD/R, astrocyte viabilities were determined by MTS assay (*n* = 3); (I) the protein levels of cleaved‐caspase3 in astrocyte were detected by WB (*n* = 4). (*p < 0.05, **p < 0.01, ***p < 0.001, ****p < 0.0001; MCAO/*R* + PBS group versus MCAO/*R* + rrTSG‐6 group, ^#^ *p* < 0.05, ^####^ *p* < 0.0001; (A) the data were expressed as median with interquartile range, Kruskal–Wallis followed by Dunnett's post hoc test was applied, (B–F, H) the data were expressed as means ± sd, one or two‐way ANOVA followed by Tukey's post hoc test was applied, (G, I) the data were expressed as median with interquartile range, Brown–Forsythe followed by Games–Howell post hoc test was applied.)

Rats were subjected to reperfusion for 12, 24, and 72 h following 2 h of MCAO. It was observed that the mRNA expression level of *Tnfaip6* (Kruskal‐Wallis statistic = 12.26, *p* = 0.0002, Figure 1A) and the protein level of TSG‐6 (*F*_(3,12)_ = 14.14, *R* ^2^ = 0.7795, *p* = 0.0003, Figure 1B) peaked at 24 h in the ischemic hemisphere after MCAO/R. Although no significant difference was detected in *Tnfaip6* mRNA expression between MCAO/R rats with or without rrTSG‐6 in the ischemic hemisphere (*F*_(2,12)_ = 6.834, *R* ^2^ = 0.5325, *p* = 0.0104, Figure S4A), TSG‐6 protein levels were found to be elevated following MCAO/R, with rrTSG‐6 treatment further enhancing TSG‐6 protein expression in the ischemic hemisphere (*F*_(2,12)_ = 26.51, *R* ^2^ = 0.8155, *p* < 0.0001, Figure S4B).

To determine the effects of rrTSG‐6 on infarction and neurobehavioral recovery in stroke rats, infarct volume was assessed using TTC staining, and neurobehavioral parameters were evaluated using the mNSS and the adhesive‐removal test. At 24 h post‐MCAO/R, TTC‐stained brain slices revealed obvious cerebral infarction, whereas rrTSG‐6 (5 μg) treatment significantly diminished infarct volumes (*F*_(2,12)_ = 274, *R* ^2^ = 0.9786, *p* < 0.0001, Figure 1C). Additionally, rrTSG‐6 treatment significantly lowered the mNSS score (Interaction *F*_(4,36)_ = 6.47, *p* = 0.0005, Row factor *F*_(2,36)_ = 24.02, *p* < 0.0001, Column Factor *F*_(2,36)_ = 458.2, *p* < 0.0001, Figure 1D) and reduced the adhesive‐removal time (Interaction *F*_(4,36)_ = 28.81, *p* < 0.0001, Row Factor *F*_(2,36)_ = 113.5, *p* < 0.0001, Column Factor *F*_(2,36)_ = 453.0, *p* < 0.0001, Figure 1E) compared with the MCAO/*R* + PBS group on days 1, 3, and 7 after MCAO/R.

Furthermore, at 24 h post‐MCAO/R, the protein levels of tight junction‐associated proteins ZO‐1 and occludin were significantly decreased, whereas MMP9 was markedly increased in the ischemic hemisphere compared with the sham group. RrTSG‐6 treatment significantly restored ZO‐1 (*F*_(2,12)_ = 48.41, *R* ^2^ = 0.8897, *p* < 0.0001, Figure 1F) and occludin (*F*_(2,12)_ = 11.5, *R* ^2^ = 0.6571, *p* = 0.0016, Figure 1F) protein levels while suppressing MMP9 (*F*_(2,12)_ = 18.53, *R* ^2^ = 0.7554, *p* = 0.0002, Figure 1F) expression compared with the MCAO/*R* + PBS group.

Apoptosis in brain tissues was quantified using the TUNEL assay. Compared with the MCAO/*R* + PBS group, rrTSG‐6 treatment significantly reduced apoptosis count in the ischemic cortex (*F*_(2.000,5.486)_ = 185.7, *p* < 0.0001, Figure 1G). Additionally, the trend of astrocyte apoptosis was consistent with the overall apoptotic pattern in the ischemic cortex, indicating that rrTSG‐6 effectively reduced astrocyte apoptosis following I/R injury (Figure 1G). In vitro experiments, primary astrocytes were cultured from postnatal day 1 SD rat pups. The morphology and identification of primary cortical astrocytes and astrocytes are shown in Figure S5. To assess the effect of rrTSG‐6 on OGD/R‐induced astrocyte viability, primary astrocytes were subjected to 6‐h OGD and subsequently treated with rrTSG‐6 at various concentrations (0, 200, 400, 600 ng/mL) for 24 h. Cell viability and apoptosis were analyzed using the MTS assay. Following reoxygenation with different rrTSG‐6 concentrations for 24 h, astrocyte viability in the OGD/*R* + PBS group significantly increased in a dose‐dependent manner, although it remained lower than that in the control group (*F*_(4,10)_ = 873.5, *R* ^2^ = 0.9971, *p* < 0.0001, Figure 1H). Based on these findings, 600 ng/mL rrTSG‐6 was selected for subsequent in vitro experiments. WB analysis demonstrated that rrTSG‐6 inhibited the OGD/R‐induced overexpression of cleaved‐caspase3, indicating a reduction in apoptotic astrocytes (*F*_(2.000,5.785)_ = 187, *p* < 0.0001, Figure 1I). In summary, rrTSG‐6 attenuated astrocyte apoptosis following acute cerebral I/R injury.

### 3.2 RrTSG‐6 Alleviates Astrocyte Reactivity and A1 Astrocyte Transformation Following Acute Cerebral I/R Injury

**FIGURE 2:**
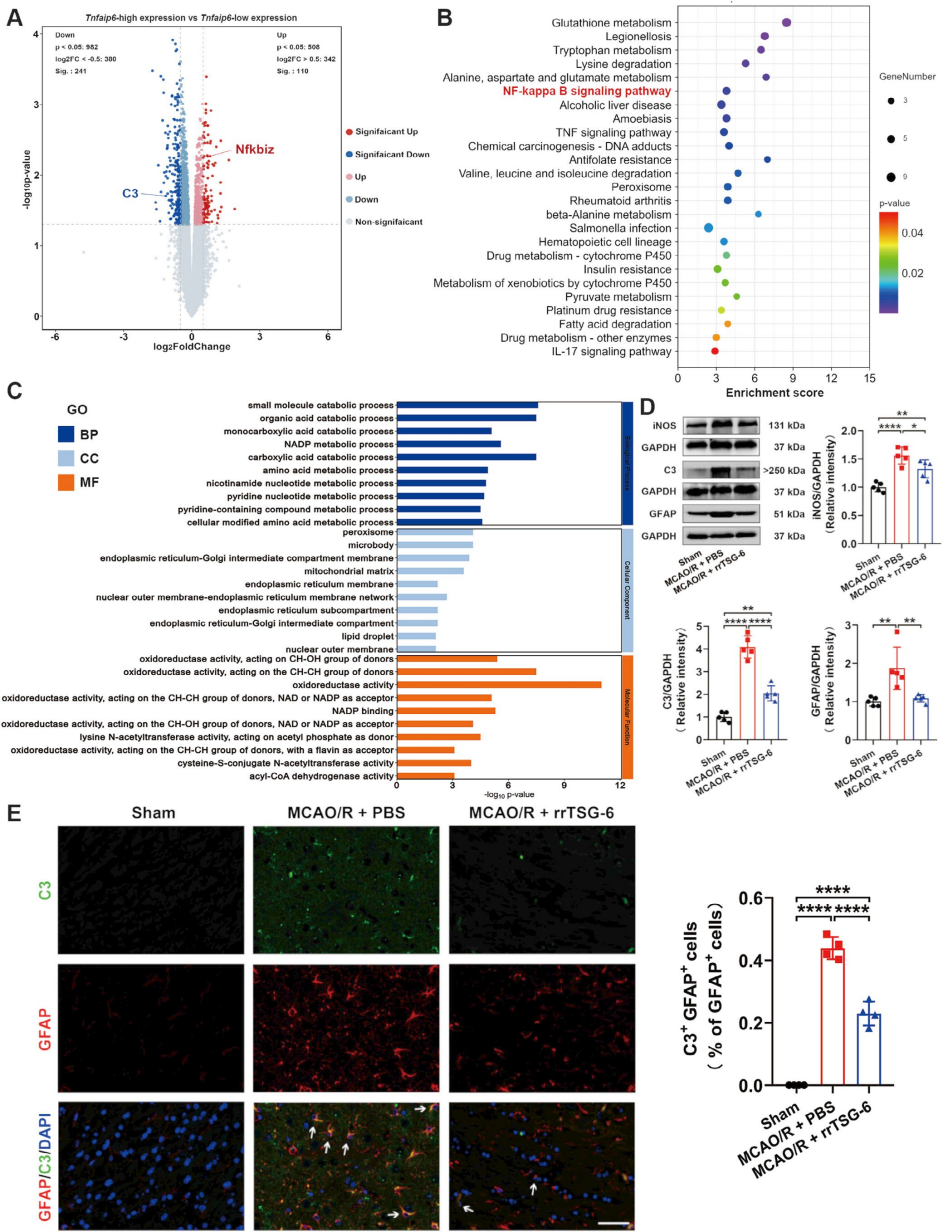
RrTSG‐6 alleviated astrocyte reactivity and A1 astrocyte transformation following acute cerebral I/R injury. (A) Volcano plot of *Tnfaip6* high/low: blue represents downregulated genes, red represents upregulated genes, and gray represents no significant differently expressed genes; (B) KEGG pathways analysis: The enriched significance gradually increases from purple to red, and the dot sizes indicate the number of differential genes contained in the corresponding pathway; (C) Enriched GO terms. At 24 h post‐MCAO/R, (D) the protein levels of iNOS, C3 and GFAP were detected by WB (*n* = 5); (E) representative photomicrographs showed C3^+^/GFAP^+^ cells in the ischemic hemisphere cortex among sham, MCAO/*R* + PBS and MCAO/*R* + rrTSG‐6 groups (*n* = 4), green represented C3, red represented GFAP, blue represented DAPI and white arrows represented C3^+^/GFAP^+^ cells, bar = 50 μm. (*p < 0.05, **p < 0.01, ****p < 0.0001, all the data were expressed as means ± sd, one‐way ANOVA followed by Tukey's post hoc test was applied.)

The normalized GSE35338 expression matrix was obtained after annotation based on the GPL1261 platform. By setting the cutoff value to *p* < 0.05 and |log2 FC| ≥ 0.5, a total of 351 DEGs, comprising 110 upregulated and 241 downregulated genes, were identified as potentially correlated with *Tnfaip6* expression in astrocytes at 24 h post‐MCAO (Figure 2A, Table S5). C3, a marker of A1 astrocytes that is exclusively expressed in A1 astrocytes but not in A2 astrocytes [20], was downregulated in the *Tnfaip6*‐high expression group, whereas *Nfkbiz*, also known as NF‐κB inhibitor zeta, was upregulated in the *Tnfaip6*‐high expression group. Subsequently, all KEGG pathway enrichment analyses and the top 10 enrichment results from BP, CC, and MF analyses, as assessed by Metascape, are presented in Figure 2B and Figure 2C. KEGG analysis indicated the NF‐kappa B signaling pathway, which is closely associated with A1 astrocyte activation [20], as the most significantly enriched signaling pathway.

**FIGURE 3:**
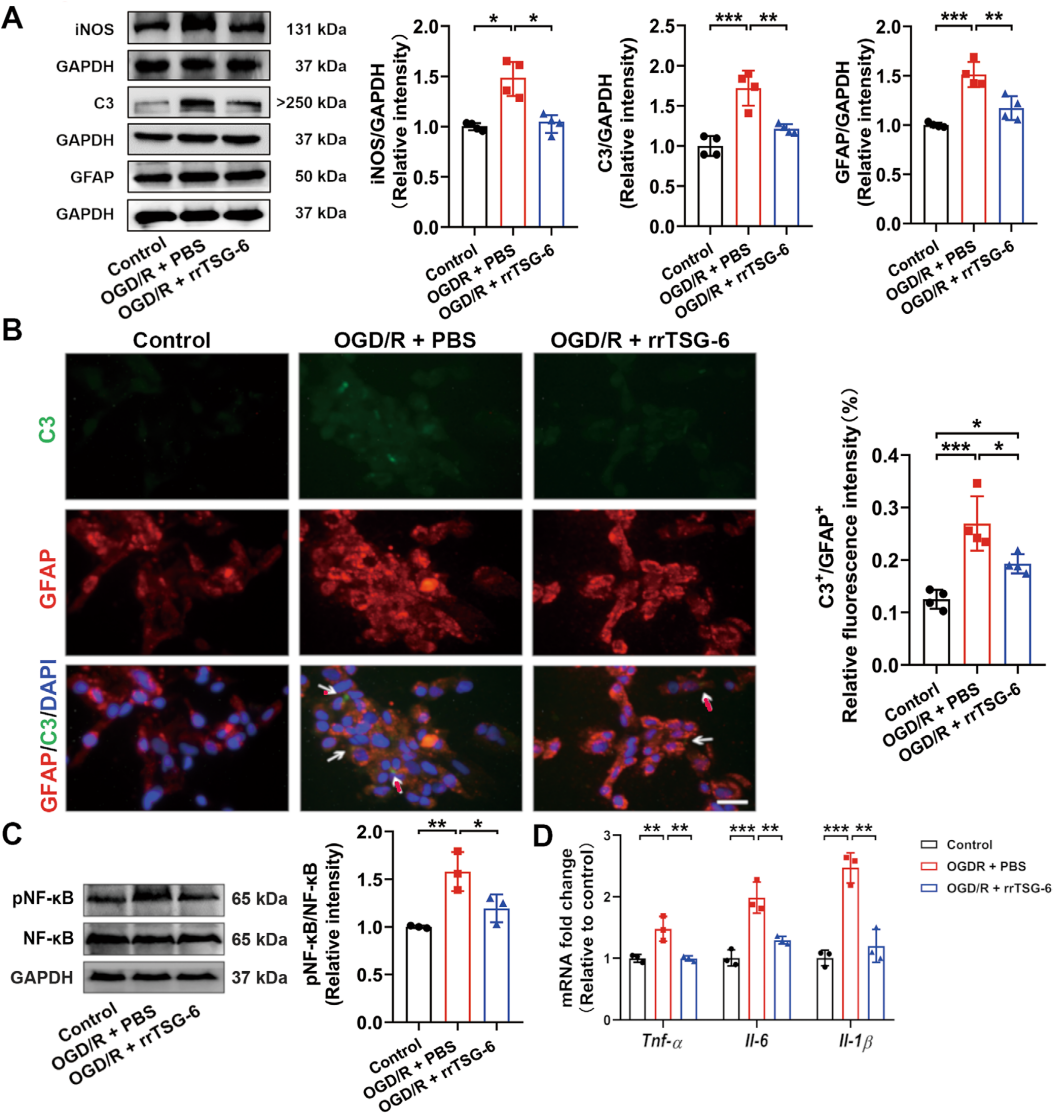
RrTSG‐6 alleviated astrocyte reactivity and A1 astrocyte transformation in OGD/R‐induced astrocytes. At 24 h post‐OGD/R, (A) the protein levels of iNOS, GFAP, and C3 (*n* = 4), and (C) phosphorylated NF‐κB levels (*n* = 3) were detected by WB; (B) astrocytes following immunofluorescence staining with antibodies against C3 and GFAP in (*n* = 4), green represented C3, red represented GFAP, blue represented DAPI, and white arrows represented C3^+^/GFAP^+^ cells, bar = 50 μm; (D) the relative mRNA fold changes of inflammatory cytokines (*Tnf‐α*, *Il‐6* and *Il‐1β*) were analyzed by RT‐qPCR (*n* = 3). (*p < 0.05, **p < 0.01, ***p < 0.001, ****p < 0.0001, **p < 0.01, ***p < 0.001, ****p < 0.0001, the protein levels of iNOS were expressed as median with interquartile range, Brown–Forsythe ANOVA followed by Games–Howell post hoc test was applied, the rest of the data were expressed as means ± sd, one‐way ANOVA followed by Tukey's post hoc test was applied.)

To determine whether TSG‐6 mitigates astrocyte reactivity and A1 astrocyte transformation following acute cerebral I/R injury, we detected the protein levels of iNOS, C3, and GFAP. We found that the protein levels of iNOS (*F*_(2,12)_ = 21.71, *R* ^2^ = 0.7835, *p* = 0.0001, Figure 2D), C3 (*F*_(2,12)_ = 92.84, *R* ^2^ = 0.9393, *p* < 0.0001, Figure 2D) and GFAP (*F*_(2,12)_ = 10.39, *R* ^2^ = 0.6340, *p* = 0.0024, Figure 2D) were significantly lower in the MCAO/*R* + rrTSG‐6 group in comparison to the MCAO/*R* + PBS group. IF staining demonstrated a reduction in C3^+^/GFAP^+^ cells, indicating a decrease in A1 astrocytes in MCAO/R rats following rrTSG‐6 treatment at 24 h post‐MCAO/R (*F*_(2,9)_ = 212.9, *R* ^2^ = 0.9793, *p* < 0.0001, Figure 2E). In an OGD/R‐induced astrocyte model, rrTSG‐6 treatment significantly decreased the protein expressions of iNOS (*F*_(2.000,4.828)_ = 19.61, *p* = 0.0048, Figure 3A), C3 (*F*_(2,9)_ = 24.8, *R* ^2^ = 0.8464, *p* = 0.0002, Figure 3A) and GFAP (*F*_(2,9)_ = 25.96, *R* ^2^ = 0.8523, *p* = 0.0002, Figure 3A) compared to the OGD/*R* + PBS group. Furthermore, rrTSG‐6 treatment inhibited the transformation of OGD/R‐induced astrocytes to the A1‐like phenotype (*F*_(2,9)_ = 18.7, *R* ^2^ = 0.8061, *p* = 0.0006, Figure 3B). To further examine NF‐κB activation, a key transcription factor regulating C3 expression in astrocytes, we measured the phosphorylation of NF‐κB and the mRNA levels of inflammatory cytokines, including *Tnf‐α*, Interleukin‐6 (*Il‐6*), and Interleukin‐1β (*Il‐1β*). In OGD/R‐induced astrocytes, phosphorylation of NF‐κB and cytokine mRNA levels were elevated, while rrTSG‐6 treatment significantly suppressed NF‐κB phosphorylation (*F*_(2,6)_ = 12.33, *R* ^2^ = 0.8043, *p* = 0.0075, Figure 3C) and reduced the mRNA levels of inflammatory cytokines (*Tnf‐α*: *F*_(2,6)_ = 15.04, *R* ^2^ = 0.8337, *p* = 0.0046; *Il‐6*: *F*_(2,6)_ = 26.85, *R* ^2^ = 0.8995, *p* = 0.001; *Il‐1β*: *F*_(2,6)_ = 38.42, *R* ^2^ = 0.9276, *p* = 0.0004; Figure 3D). Collectively, these findings indicate that rrTSG‐6 treatment alleviates astrocyte reactivity and A1 astrocyte transformation following acute cerebral I/R injury.

### 3.3 Effect of rrTSG‐6 on Hsp70 Family‐Related Genes in OGD/R‐Induced Astrocytes In Vitro

After quality screening of sample data and exclusion of non‐coding genes and pseudogenes, a total of 21,684 coding genes were selected for subsequent analysis (Figure S3, Additional file 2). Using |log2FC| ≥ 0.5 and adj. *p* < 0.05 as the threshold, 418 DEGs were identified, including 24 upregulated genes and 380 downregulated genes in the OGD/*R* + PBS group compared to the control group (Table S6). A heatmap illustrating the expression of the top 50 DEGs ranked by absolute fold change is presented in Figure S6A. The 418 DEGs were analyzed using the Metascape and KOBAS‐i platforms. The intersection of GO analysis results from both tools revealed 344 common enrichment terms, including 208 BP, 82 CC, and 54 MF enrichment terms (Figure S6B). Furthermore, 10 common enriched pathways were identified through KEGG analysis (Figure S6C). The top 10 enriched BP, CC, and MF terms, along with all KEGG‐enriched pathways identified by both Metascape and KOBAS‐i tools, are shown in Figure S6D,E. These findings suggest that OGD/R‐induced astrocytes were affected at multiple stages of cell cycle regulation.

**FIGURE 4:**
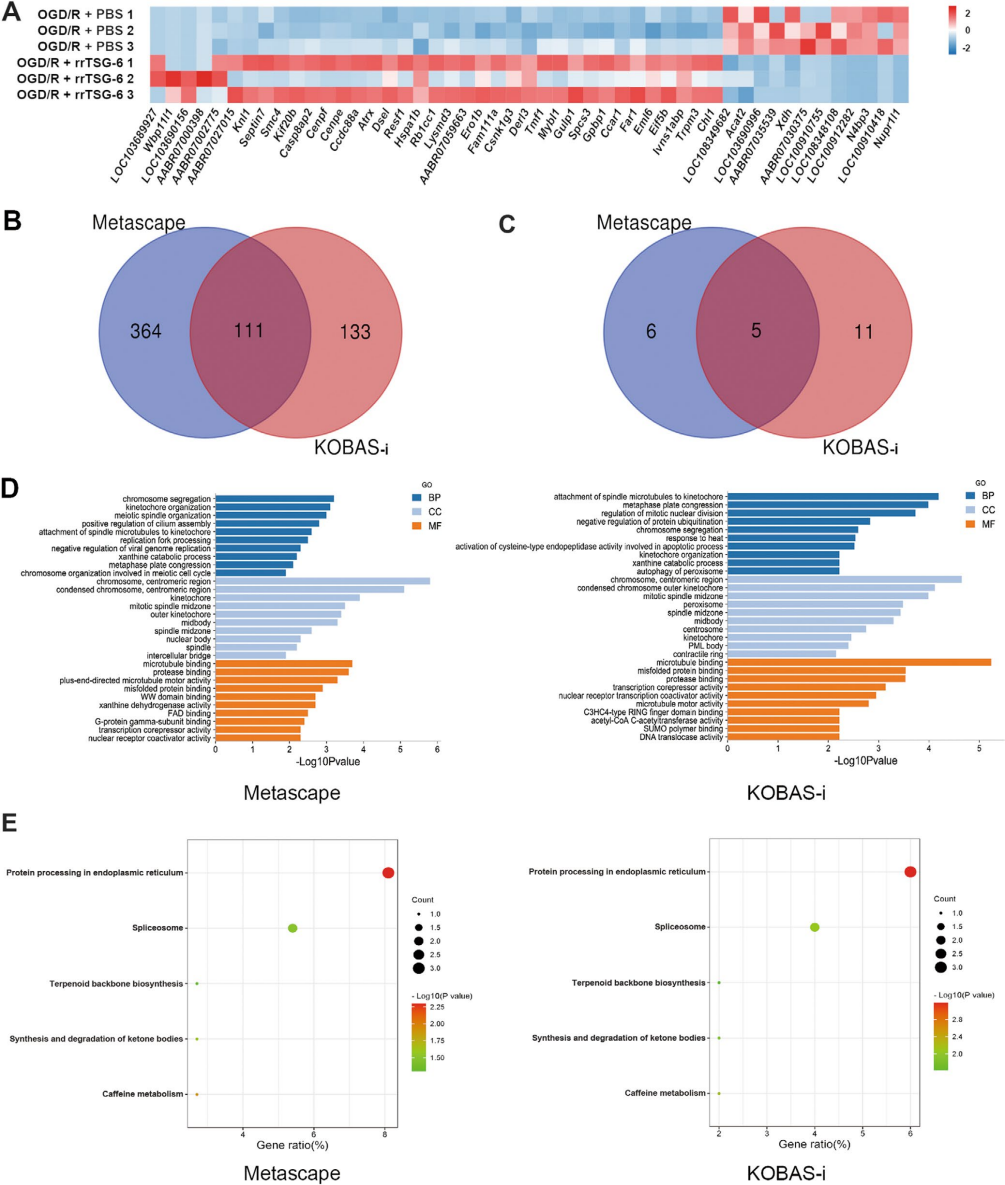
Differential expression between OGD/*R* + PBS and OGD/*R* + rrTSG‐6 groups. (A) DEGs between OGD/*R* + PBS and OGD/*R* + rrTSG‐6 groups; venn diagram of the intersection of DEGs enrichment analysis results respectively performed by (B) GO and (C) KEGG enrichment analysis based on both Metascape and KOBAS‐i; (D) top 10 enriched GO terms in BP, CC and MF sections respectively using Metascape and KOBAS‐i; (E) KEGG pathway analysis respectively using Metascape and KOBAS‐i: The enriched significance gradually increases from green to red, and the dot sizes indicate the number of DEGs contained in the corresponding pathway.

Comparing the OGD/*R* + rrTSG‐6 group to the OGD/*R* + PBS group, 49 DEGs were identified, including 12 downregulated genes and 37 upregulated genes (Table S7). In Figure 4A, the DEGs heatmap is shown in Figure 4A. GO analysis using Metascape and KOBAS‐i yielded 475 and 244 enrichment results, respectively, while KEGG analysis identified 11 and 16 enrichment pathways (Figure 4B,C). Intersection of the KEGG and GO enrichment results revealed 111 common GO terms (61 BP, 22 CC, and 28 MF) and five common KEGG pathways (Figure 4B,C). The top 10 enriched BP, CC, and MF terms from Metascape and KOBAS‐i are shown in Figure 4D. Among the KEGG‐enriched pathways identified, protein processing in the endoplasmic reticulum (ER) was ranked highest in both platforms (Figure 4E).

**FIGURE 5:**
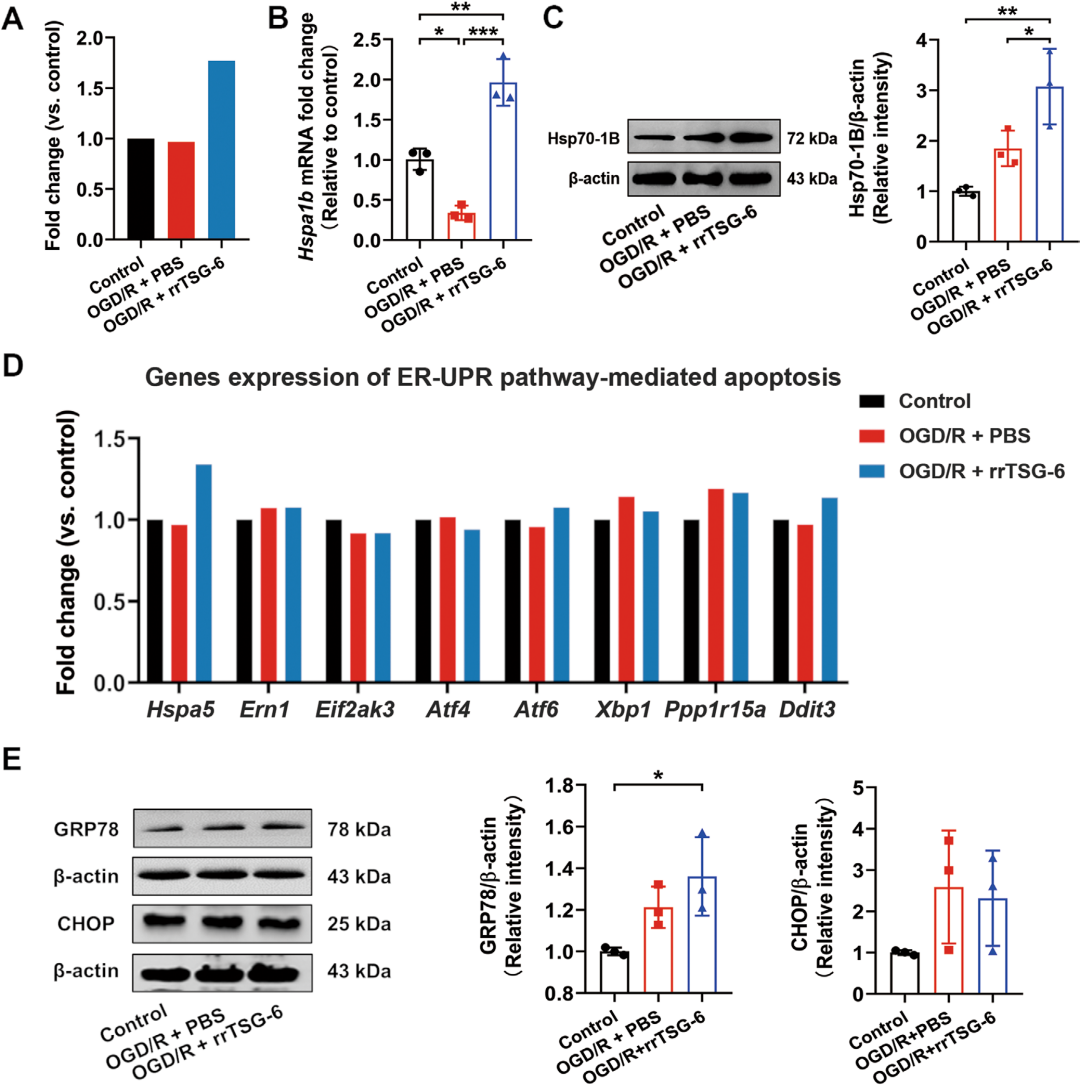
Effect of rrTSG‐6 on Hsp70 family related genes in OGD/R‐induced astrocytes. At 24 h post‐OGD/R, the relative *Hspa1b* mRNA fold changes were analyzed by (A) mRNA‐seq and (B) RT‐qPCR (*n* = 3); (C) Hsp70‐1B protein levels were detected by WB (*n* = 3); (E) genes expressions of ERS pathway‐mediated apoptosis were analyzed by mRNA‐seq (*n* = 3). (F) GRP78 and CHOP protein levels were detected by WB (*n* = 3). (*p < 0.05, **p < 0.01, ***p < 0.001, all the data were expressed as means ± sd, one‐way ANOVA followed by Tukey's post hoc test was applied.)

Among the genes enriched in the pathway of protein processing in the ER, the *Hspa1b* gene exhibited the most significant fold change. Based on mRNA‐seq, rrTSG‐6 upregulated the transcription level of the *Hspa1b* gene in OGD/R‐induced astrocytes. However, compared to the control group, no obvious difference was observed in the transcription level of the *Hspa1b* gene in OGD/R‐induced astrocytes (Figure 5A). Further verification through RT‐qPCR revealed that, in comparison to the control group, the mRNA level of *Hspa1b* was significantly decreased in OGD/R‐induced astrocytes, while rrTSG‐6 treatment significantly increased *Hspa1b* expression (*F*_(2,6)_ = 54.55, *R* ^2^ = 0.9479, *p* = 0.0001, Figure 5B). WB analysis revealed that Hsp70‐1B, a member of the Hsp70 family encoded by *Hspa1b*, was overexpressed at the protein level in the OGD/*R* + rrTSG‐6 group compared to both the OGD/*R* + PBS and the control groups. In contrast to the control group, astrocytes in the OGD/*R* + PBS group exhibited a noteworthy reduction in the mRNA levels of *Hspa1b*, whereas its encoded protein showed no significant differences between these two groups (*F*_(2,6)_ = 14.12, *R* ^2^ = 0.8247, *p* = 0.0054, Figure 5C).

Based on the enrichment analysis of DEGs in OGD/R‐induced astrocytes with or without rrTSG‐6 treatment, a potential mechanism known as protein processing in the ER was identified. Consequently, our attention was directed toward GRP78, encoded by the *Hspa5* gene. Notably, GRP78 is the only protein within the Hsp70 family that is localized in the ER [38]. The potential involvement of ER stress (ERS) in apoptosis was considered. However, mRNA‐seq analysis showed no significant differences in the transcription levels of *Hspa5* and other genes related to ERS‐mediated apoptosis between the OGD/*R* + PBS and the OGD/*R* + rrTSG‐6 groups (Figure 5D). Further detection of the protein levels of GRP78 and CHOP, key proteins involved in the ERS‐mediated apoptosis pathway, showed that the GRP78 protein expression in the OGD/*R* + rrTSG‐6 group was significantly higher than in the control group, but not significantly different from that of the OGD/*R* + PBS group (*F*_(2,6)_ = 6.438, *R* ^2^ = 0.6821, *p* = 0.0321, Figure 5E). No significant differences were observed in CHOP protein levels among the three groups (*F*_(2,6)_ = 2.028, *R* ^2^ = 0.4034, *p* = 0.2124, Figure 5E).

### 3.4 RrTSG‐6 Alleviated OGD/R‐Induced Astrocyte Apoptosis, Astrocyte Reactivity, and A1 Astrocyte Transformation via Upregulation of Hsp70‐1B In Vitro

**FIGURE 6:**
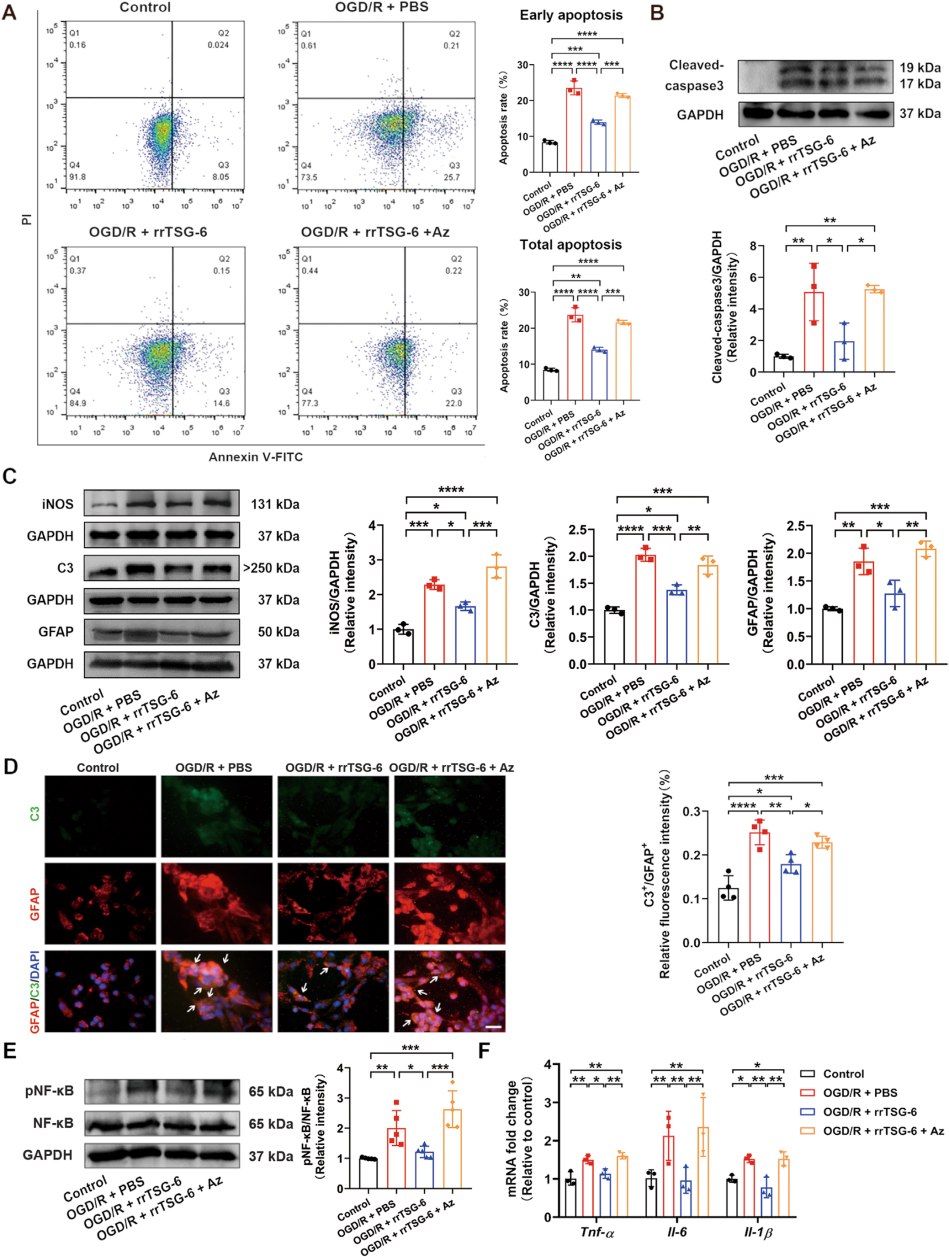
RrTSG‐6 alleviated OGD/R‐induced astrocyte apoptosis, reactivity, and A1 astrocyte transformation via upregulating Hsp70‐1B. (A) Astrocyte apoptosis was measured by flow cytometry. Q1, Q2, Q3, and Q4 respectively represent quadrants for dead, late apoptotic, early apoptotic and viable cells (*n* = 3). The protein levels of (B) cleaved‐caspase3 (*n* = 3), (C) iNOS, C3 and GFAP (*n* = 3), and (E) phosphorylated NF‐κB levels (*n* = 5) in astrocyte were detected by WB at 24 h post‐OGD/R. (D) Astrocytes following immunofluorescence staining with antibodies against C3 and GFAP (*n* = 4), green represented C3, red represented GFAP, blue represented DAPI and white arrows represented C3^+^/GFAP^+^ cells, bar = 50 μm; (F) the relative mRNA fold changes of inflammatory cytokines (*Tnf‐α*, *Il‐6* and *Il‐1β*) in astrocytes were analyzed by RT‐qPCR at 24 h post‐OGD/R (*n* = 3). (*p < 0.05, **p < 0.01, ***p < 0.001, ****p < 0.0001, all the data were expressed as means ± sd, one‐way ANOVA followed by Tukey's post hoc test was applied.)

To investigate the role of Hsp70‐1B, a Hsp70‐1B inhibitor, Az, was administered at concentrations of 0, 5, 10, and 15 μmol/L in OGD/R‐induced astrocytes treated with rrTSG‐6. A concentration‐dependent decrease in astrocyte viability was observed, indicating that Az counteracted the beneficial effects of rrTSG‐6 on the cell viability of OGD/R‐induced astrocytes (Figure S7). At 24 h post‐OGD/R, both early apoptosis and total apoptosis rates were significantly increased compared to the control group. RrTSG‐6 significantly inhibited early apoptosis in OGD/R‐induced astrocytes, whereas Az abrogated the effect of rrTSG‐6 on both early apoptosis and total apoptosis rates (early apoptosis: *F*_(3,8)_ = 125.5, *R* ^2^ = 0.9792, *p* < 0.0001, Figure 6A; total apoptosis: *F*_(3,8)_ = 123.5, *R* ^2^ = 0.9789, *p* < 0.0001, Figure 6A).

The protein levels of cleaved‐caspase3, iNOS, C3, GFAP, and phosphorylated NF‐κB and the mRNA levels of inflammatory cytokines (*Tnf‐α*, *Il‐6* and *Il‐1β*) in the control, OGD/*R* + PBS, and OGD/*R* + rrTSG‐6 groups changed consistent with previous findings. Az administration significantly inhibited the effect of rrTSG‐6 on the protein levels of cleaved‐caspase3 (*F*_(3,8)_ = 11.95, *R* ^2^ = 0.8176, *p* = 0.0025, Figure 6B), iNOS (*F*_(3,8)_ = 44.51, *R* ^2^ = 0.9435, *p* < 0.0001, Figure 6C), C3 (*F*_(3,8)_ = 46.84, *R* ^2^ = 0.9461, *p* < 0.0001, Figure 6C), GFAP (*F*_(3,8)_ = 22.46, *R* ^2^ = 0.8939, *p* = 0.0003, Figure 6C) and phosphorylated NF‐κB (*F*_(3,16)_ = 15.02, *R* ^2^ = 0.7379, *p* < 0.0001, Figure 6E) and the mRNA levels of inflammatory cytokines (*Tnf‐α*: *F*_(3,8)_ = 15.09, *R* ^2^ = 0.8498, *p* = 0.0012; *Il‐6*: *F*_(3,8)_ = 17.47, *R* ^2^ = 0.8675, *p* = 0.0007; *Il‐1β*: *F*_(3,8)_ = 13.77, *R* ^2^ = 0.8378, *p* = 0.0016; Figure 6F) in OGD/R‐induced astrocytes. Additionally, Az suppressed the transformation of OGD/R‐induced astrocytes into A1‐like astrocytes (*F*_(3,12)_ = 23.07, *R* ^2^ = 0.8522, *p* < 0.0001, Figure 6D). These findings suggest that rrTSG‐6 alleviates OGD/R‐induced astrocyte apoptosis, astrocyte reactivity, and A1 astrocyte transformation through the upregulation of Hsp70‐1B in vitro.

## 4 Discussion

Previous studies have demonstrated the anti‐inflammatory and neuroprotective effects of TSG‐6 in various neurological disorders [1]. The present findings indicate that rrTSG‐6 exerts a protective effect against I/R injury by enhancing neurological function, reducing infarct volume, and preserving the BBB integrity. This protective role in the BBB has been previously documented in rat models of ICH and SAH [5, 6, 7]. Furthermore, the current study revealed the anti‐apoptotic properties of rrTSG‐6 in cerebral infarction in rat models. In vitro experiments demonstrated a concentration‐dependent increase in astrocyte viability in OGD/R‐induced astrocytes treated with rrTSG‐6, further substantiating its anti‐apoptotic effect in cerebral I/R injury. These findings are consistent with those of Tang et al. [6], who reported similar effects of TSG‐6 in rat models of SAH and LPS‐induced astrocyte injury.

To further investigate the potential effects of TSG‐6 on astrocytes in ischemic brain tissue, transcriptomic data of astrocytes sorted by magnetic beads following MCAO/R were analyzed using the GEO database. The results revealed that in the *Tnfaip6*‐high expression group, the A1 astrocyte marker C3 was downregulated, whereas the NF‐κB inhibitor gene, *Nfkbiz*, was upregulated, compared to the *Tnfaip6*‐low expression group. KEGG pathway analysis further indicated that the NF‐κB signaling pathway, closely associated with A1 astrocytes, was the most significantly enriched pathway. Additionally, previous studies have demonstrated that modulating A1 astrocyte polarization is a critical therapeutic target for acute IS. For instance, AD16 has been shown to mitigate ischemic stroke‐induced brain injury by reducing A1 astrocyte polarization, thereby suppressing neuroinflammation via the downregulation of NF‐κB and Janus Kinase 2 (JAK2)/signal transducer and activator of transcription 3 (STAT3) signaling pathway [39]. Furthermore, astrocyte‐derived Krüppel‐like factor 4 plays a critical role in regulating A1/A2 astrocyte activation following acute IS by modulating NF‐κB activation [40]. These findings underscore the significance of astrocyte phenotype regulation in IS and highlight the protective effects of TSG‐6 and other therapeutic targets on IS outcomes.

The present study showed increased expression levels of GFAP, C3, and an inflammatory marker iNOS in the infarct hemisphere at 24 h post‐MCAO/R, as well as astrocytes at 24 h post‐OGD/R. A substantial increase in GFAP expression largely reflects astrocyte cytoskeletal changes, which indirectly indicate astrocyte reactivity [41]. C3, a secretory complement factor, recently gained recognition as a marker of both astrocyte reactivity and the A1 phenotype in reactive astrocytes [20, 42]. These observations suggest an increase in astrocyte reactivity and A1 polarization following acute cerebral I/R injury. Previous studies have reported that TSG‐6 gene knockout mice exhibited heightened astrocyte reactivity in and around the injury site, implying a potential anti‐inflammatory role of TSG‐6 in glial scar formation [23], thereby indirectly supporting the findings of the present study. In a transient MCAO model, Zhang et al. [43] reported that with prolonged reperfusion time, the number of C3^+^/GFAP^+^ astrocytes surrounding the lesion increased and continued to rise up to 14 days post‐reperfusion, while the number of S100 calcium binding protein A10 (S100A10)^+^/GFAP^+^ cells gradually declined. These findings suggest that alterations in A1 and A2 astrocyte phenotypes occur following cerebral I/R injury, with the A2 phenotype predominating in the early reperfusion phase, whereas the A1 phenotype, associated with immune and inflammatory functions, increases over time. The present study further demonstrated that exogenous rrTSG‐6 attenuated excessive astrocyte reactivity and the proinflammatory A1 phenotype transformation. To the best of our knowledge, this is the first study to elucidate the effects of TSG‐6 on astrocyte phenotypes and the underlying regulatory mechanisms.

Subsequently, mRNA‐seq analysis was performed, revealing that intracellular alteration in OGD/R‐induced astrocytes was primarily associated with cell cycle regulation and stress responses. Comparative analysis of OGD/R‐induced astrocytes with or without rrTSG‐6 treatment identified 49 DEGs. Enriched GO and KEGG pathways analyses of these DEGs indicated their involvement in cell cycle regulation, apoptosis, autophagy, ER protein processing, and other biological processes. Notably, protein processing in the ER emerged as the most significantly enriched pathway in KEGG enrichment analysis across both platforms. The ER plays a critical role in protein synthesis and processing, thereby regulating proteostasis [44]. The unfolded protein response (UPR) is a signal transduction network that mitigates ER stress and coordinates protein homeostasis to maintain cell viability [45]. Proteostasis, which governs protein processing, folding, quality control, and degradation, is fundamental to astrocyte function and is implicated in various neurological disorders [44]. The astrocyte reactivity state resulting from UPR dysregulation in the ER may represent a viable therapeutic target for neurological diseases [46]. Among the genes within this pathway, *Hspa1b* exhibited the most substantial fold change. Its encoded protein, Hsp70‐1B, belongs to the Hsp70 family, one of the most highly conserved protein families across all living organisms. Hsp70 family proteins function to prevent protein aggregation and facilitate proper protein folding [47]. In rodents, the Hsp70 family protein consists of at least seven isoforms, among which only Hsp70‐1A (Hsp70.3)/Hsp70‐1B (Hsp70.1) and glucose‐regulated protein 78 (GRP78/HspA5) are inducible under ischemic conditions, heat shock, and other stress stimuli [48, 49, 50].

However, the present mRNA‐seq analysis demonstrated no significant difference in *Hspa1b* transcription in OGD/R‐induced astrocytes compared to the control group. This finding was further validated at both the mRNA and protein levels through RT‐qPCR and WB, respectively. In contrast to the control group, the astrocytes in the OGD/*R* + PBS group exhibited a significant reduction in *Hspa1b* mRNA level, whereas the encoded protein showed no significant difference between the two groups. These results align with previous research indicating that Hsp70‐1B protein levels in astrocytes increase with prolonged OGD duration, peaking at 12 h [51]. However, upon reperfusion, Hsp70‐1B protein levels decline progressively at 0, 12, and 24 h, with levels at 24 h post‐OGD/R potentially lower than those in the control group [52]. Multiple studies have corroborated that increased Hsp70‐1B expression in ischemic brain tissues confers neuroprotection. For instance, upregulation of Hsp70‐1B has been reported to stabilize the lysosomal membrane of OGD/R‐induced astrocytes, a process closely associated with early apoptosis [51, 53, 54]. Moreover, valproic acid has been shown to mitigate astrocyte reactivity after OGD/R by upregulating Hsp70‐1B expression [52]. The present study demonstrated that rrTSG‐6 inhibited early apoptosis, reduced astrocyte reactivity, suppressed the A1 phenotype, and upregulated Hsp70‐1B expression in OGD/R‐induced astrocytes. Consequently, it is proposed that the increased expression of Hsp70‐1B may play a critical role in the neuroprotective effects of rrTSG‐6 in OGD/R‐induced astrocytes.

To further validate this hypothesis, an in vitro study was conducted using a combination treatment of rrTSG‐6 and the Hsp70‐1B inhibitor, Az. Az, an apoptosis inducer, primarily facilitates lysosomal membrane permeability by translocating into lysosomes, thereby promoting lysosomal‐mediated apoptosis [54]. Our results showed that the Az intervention counteracted the anti‐apoptotic effects of rrTSG‐6 and its ability to attenuate excessive astrocyte reactivity and transformation into the A1 phenotype. Previous studies have indicated that TSG‐6 mitigated early apoptosis in astrocytes stimulated by LPS via inhibiting the Toll‐like receptor 1/2‐NF‐κB canonical pathway [5, 7]. Meanwhile, NF‐κB, a key factor in neuroinflammation, has been implicated in the regulation of astrocyte reactivity and phenotypic shifts. For instance, dopamine has been reported to mediate neuroprotection by blocking NF‐κB and NOD‐like receptor thermal protein domain associated protein 3‐induced A1 phenotype transformation [55]. Additionally, exosomes derived from MSCs have been shown to suppress A1 astrocytes by downregulating NF‐κB phosphorylation in spinal cord injury (SCI) [56]. Consistent with these findings, our study revealed that rrTSG‐6 reduced NF‐κB phosphorylation in OGD/R‐induced astrocytes.

Given the observed effects of rrTSG‐6 on astrocyte apoptosis, reactivity, Hsp70‐1B expression, and NF‐κB phosphorylation in this study, in conjunction with previous research indicating that both Hsp70‐1B and NF‐κB regulate astrocyte apoptosis and reactivity, we hypothesized a potential regulatory relationship between these two factors. Notably, we found that Az reversed the inhibitory effect of rrTSG‐6 on NF‐κB phosphorylation. Previous studies have reported a similar regulatory relationship, as evidenced by findings in sterile alpha and TIR motif‐containing 1 conditional knockout mice, in which neuroinflammation during the early phase of SCI was alleviated via downregulation of NF‐κB signaling, potentially due to the upregulation of *Hspa1a* and *Hspa1b* [57]. Furthermore, Hsp70 has been demonstrated to modulate NF‐κB activation through inhibiting IκB‐α phosphorylation or acting as a chaperone molecule to prevent NF‐κB binding to target genes by facilitating its degradation in alveolar macrophages of patients with active pulmonary tuberculosis [58]. However, the interplay between Hsp70‐1B and NF‐κB varies depending on physiological conditions. For instance, in a mouse model of myocardial I/R injury, ischemic preconditioning (4 min ischemia and 4 min reperfusion) with the first 24 h following 30‐min coronary occlusion and 24‐h reperfusion resulted in *Hspa1a* and *Hspa1b* being the most significantly regulated NF‐κB‐dependent genes. Notably, Hsp70‐1A (*Hspa1a*) exhibited protective effects, whereas Hsp70‐1B (*Hspa1b*) exacerbated myocardial injury. However, subsequent studies using knockout mice indicated that Hsp70‐1B contributed to NF‐κB‐dependent cardioprotection following permanent coronary occlusion, suggesting that Hsp70‐1B may exert differential effects depending on the nature of the ischemic stimuli [59, 60]. Based on these findings, we propose that TSG‐6 exerts its protective effects against astrocyte apoptosis under ischemic and hypoxic conditions by up‐regulating Hsp70‐1B and inhibiting NF‐κB phosphorylation in vitro.

Notably, KEGG enrichment analysis of DEGs between the OGD/*R* + PBS group and the OGD/*R* + rrTSG‐6 group identified protein processing in the ER as the most significantly enriched pathway. This finding led us to investigate whether ER stress‐mediated apoptosis might be a potential underlying mechanism. The stress‐inducible Hsp70 protein GRP78, encoded by *Hspa5*, is the only ER‐localized isoform of the Hsp70 family and plays a crucial role in the ERS‐mediated apoptosis pathway [47]. Upon rrTSG‐6 treatment, mRNA‐seq analysis revealed an upward trend in *Hspa5* expression in OGD/R‐induced astrocytes compared to untreated astrocytes. However, no substantial differences were observed in GRP78 protein levels between the OGD/*R* + PBS and the OGD/*R* + rrTSG‐6 groups. Additionally, genes associated with ERS‐mediated apoptosis exhibited no clear patterns of expression alterations among the three groups. In particular, no significant differences were observed in CHOP, a key protein involved in ERS‐mediated apoptosis. Based on these findings, we hypothesize that apoptosis at this time point is not significantly influenced by ERS and that ERS‐mediated apoptosis may occur at an earlier stage of reperfusion. Notably, a previous study confirmed that TSG‐6 mitigates neuron damage in cerebral IR injury via the inhibition of ERS‐related inflammatory responses [11]. These observations suggest that TSG‐6 may play a regulatory role in the ERS process at the earlier stage of OGD/R‐induced astrocyte injury. However, further research is required to substantiate this hypothesis.

This study has certain limitations. First, prior studies have shown that TSG‐6 effectively inhibits the conversion of microglia into the pro‐inflammatory M1 subtype in various neurological disorders. Furthermore, M1 microglia have been shown to produce TNF‐α, IL‐1α and complement component 1q, all of which promote A1 astrocyte transformation in certain CNS diseases [20]. Our findings indicate that the changes in C3 levels were more pronounced in vivo, and the protective effects of TSG‐6 were also more significant in vivo compared to in vitro. This suggests that TSG‐6 may not act solely on astrocytes but may also hinder A1 astrocyte transformation by preventing the polarization of microglia into subtype M1 in the rat model of cerebral I/R injury. Numerous studies have highlighted the existence of intricate crosstalk mechanisms among astrocytes, microglia, neurons, oligodendrocytes, and other CNS cell types. However, these interactions warrant further investigation. Second, astrocyte reactivity is a prolonged and dynamic process. This study focused on astrocyte reactivity in the acute phase of cerebral I/R, while astrocyte responses in the subacute and chronic phases were not examined. TSG‐6 has been reported to exert protective effects in chronic models of other systemic diseases [1]. Further research is necessary to explore its role in the recovery period of acute cerebral ischemia or chronic cerebral ischemia models. Lastly, an important consideration is whether TSG‐6, as an exogenous recombinant protein with protective properties, can be translated into a therapeutic agent for human diseases, which requires further investigation.

## 5 Conclusion

Our findings demonstrate that TSG‐6 exerts neuroprotective effects by upregulating Hsp70‐1B to inhibit NF‐κB phosphorylation in OGD/R‐induced astrocytes, thereby suppressing apoptosis, astrocyte reactivity, and A1 phenotype transformation following acute ischemic stroke. Nevertheless, further studies are warranted to elucidate the long‐term therapeutic potential of TSG‐6, which may provide a good foundation for drug development and offer new therapeutic prospects for patients with IS.

## Author Contributions

Yewei Qu: conceptualization, data curation, writing – original draft, software, validation, and funding acquisition. Lian Yi, Yushi Tang, and Fan Yang: data curation and validation. Byron Fei Pan: software, validation, and writing – review. Shanshan Shi, Changda Qu, and Fangqin Li: software and validation. Shirong Wen: writing – review and supervision. Yujun Pan: conceptualization, writing – review, supervision, and funding acquisition. All authors have read and agreed to the published version of the manuscript.

## Ethics Statement

All the animal experiments were approved by the First Affiliated Hospital of Harbin Medical University, Institutional Animal Care and Use Committee (IACUC application: 2019050) and carried out in accordance with the guidelines of the National Institutes of Health for the care and use of laboratory animals. There are no human subjects or tissues used in this study.

## Consent

The authors have nothing to report.

## Conflicts of Interest

The authors declare no conflicts of interest.

## Supporting information


Data S1.

## Data Availability

The data that support the findings of this study are available from the corresponding author upon reasonable request.
