# Supplementary material for: TSG‐6 Protects Against Cerebral Ischemia–Reperfusion Injury via Upregulating Hsp70‐1B in Astrocytes
Source: CNS Neurosci Ther. 2025 Mar 25;31(3):e70354. doi: 10.1111/cns.70354 (PMC11933850; doi:10.1111/cns.70354)
Supplement: Supplementary file 1 — Data S1. [file CNS-31-e70354-s001.doc]

**
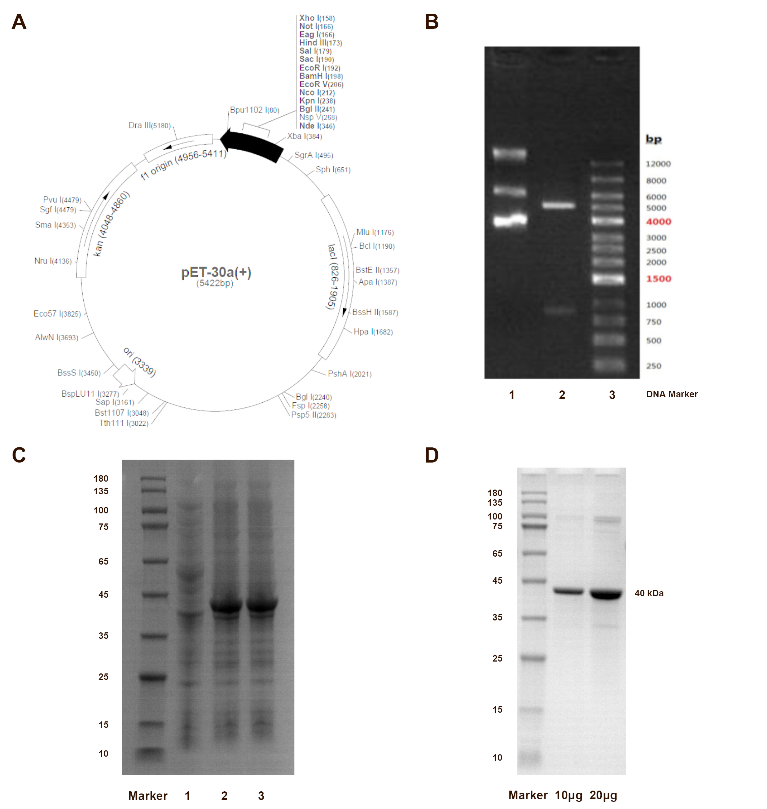
**

**Fig.S1. rrTSG-6 protein synthesis. (A)** pET-30a(+) vector map. **(B)** Plasmid and pET-30a-Tnfaip6 identification of restriction enzyme digestion. Lane 1 represented plasmid, lane 2 represented pET-30a-*Tnfaip6*. **(C)** rrTSG-6 expression analysis. Lane 1 represented rrTSG-6 with no IPTG induction, lane 2 represented rrTSG-6 with 0.50.5 mmol/L IPTG and 17 ℃ induction, lane 3 represented rrTSG-6 with 0.5 mmol/L IPTG and 37 ℃ induction. **(D)** rrTSG-6 purification and identification. (Molecular weight 40 kDa, purity > 90%).


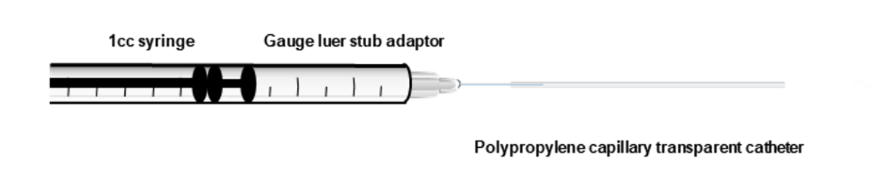


**Fig.S2 Injection unit.** Injection unit consists of a 31G insulin needle and a polypropylene catheter with 0.25 mm inner diameter and 0.35 mm outer diameter.

**
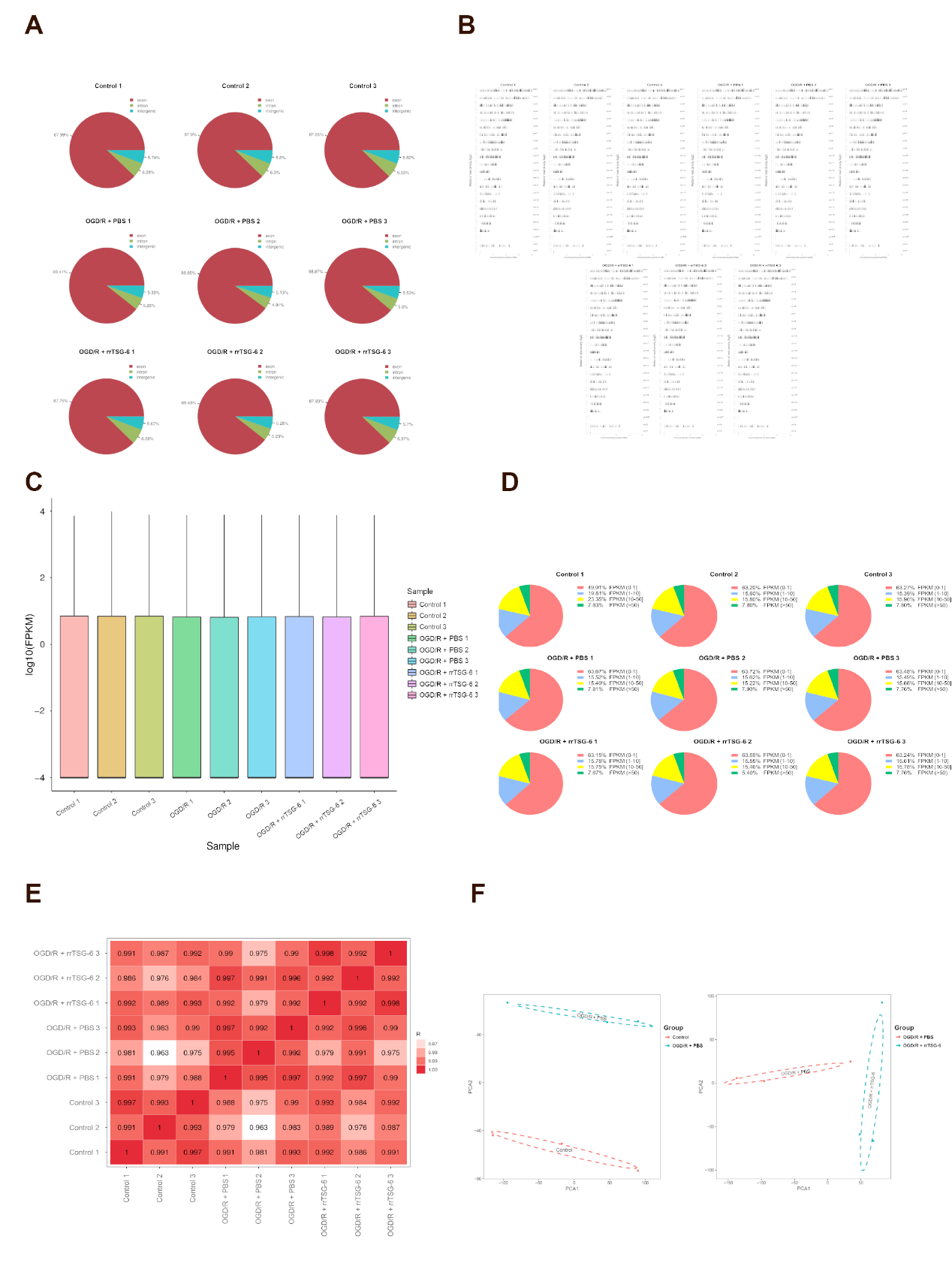
**

**Fig.S3 Data quality control. (A)** Area distribution of reads compared with reference genome sequences. **(B)** Density distribution on chromosomes of reads compared with reference genome sequences. **(C)** Distribution of FPKM in each sample. **(D)** The proportion of genes at different expression levels in each sample. **(E)** Pearson correlation analysis among samples. **(F)** Principal component analysis.


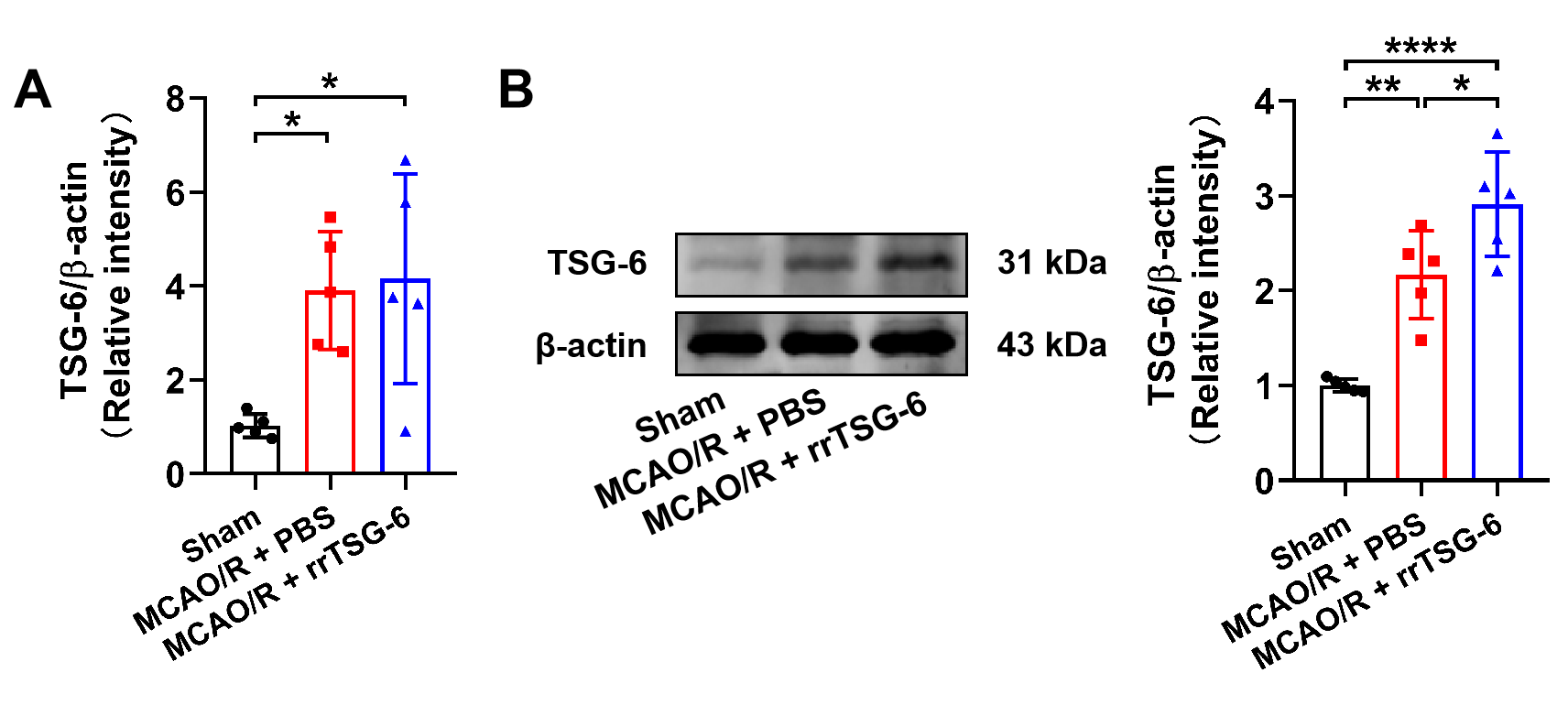


**Fig.S4 TSG-6 expression following MCAO/R with and without rrTSG-6 treatment.** **(A)** mRNA levels of *Tnfaip6* and **(B)** protein levels of TSG-6 were detected respectively by RT-qPCR and WB at 24 h following MCAO/R (n = 5). (^*^*P* < 0.05, ^**^*P* < 0.01, ^****^*P* < 0.0001, all the data were expressed as means ± sd, one-way ANOVA followed by Tukey's post hoc test was applied.)


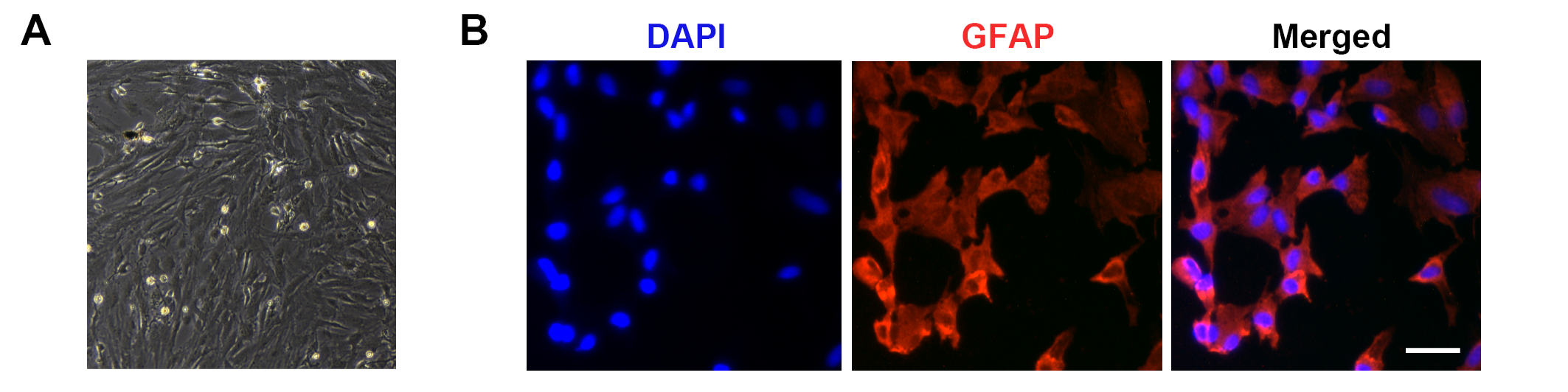


**Fig.S5 Culture and identification of primary astrocytes. (A)** Morphology under light microscope. **(B)** GFAP immunofluorescence identification, red represents GFAP and blue represents DAPI. Bar = 100 μm.


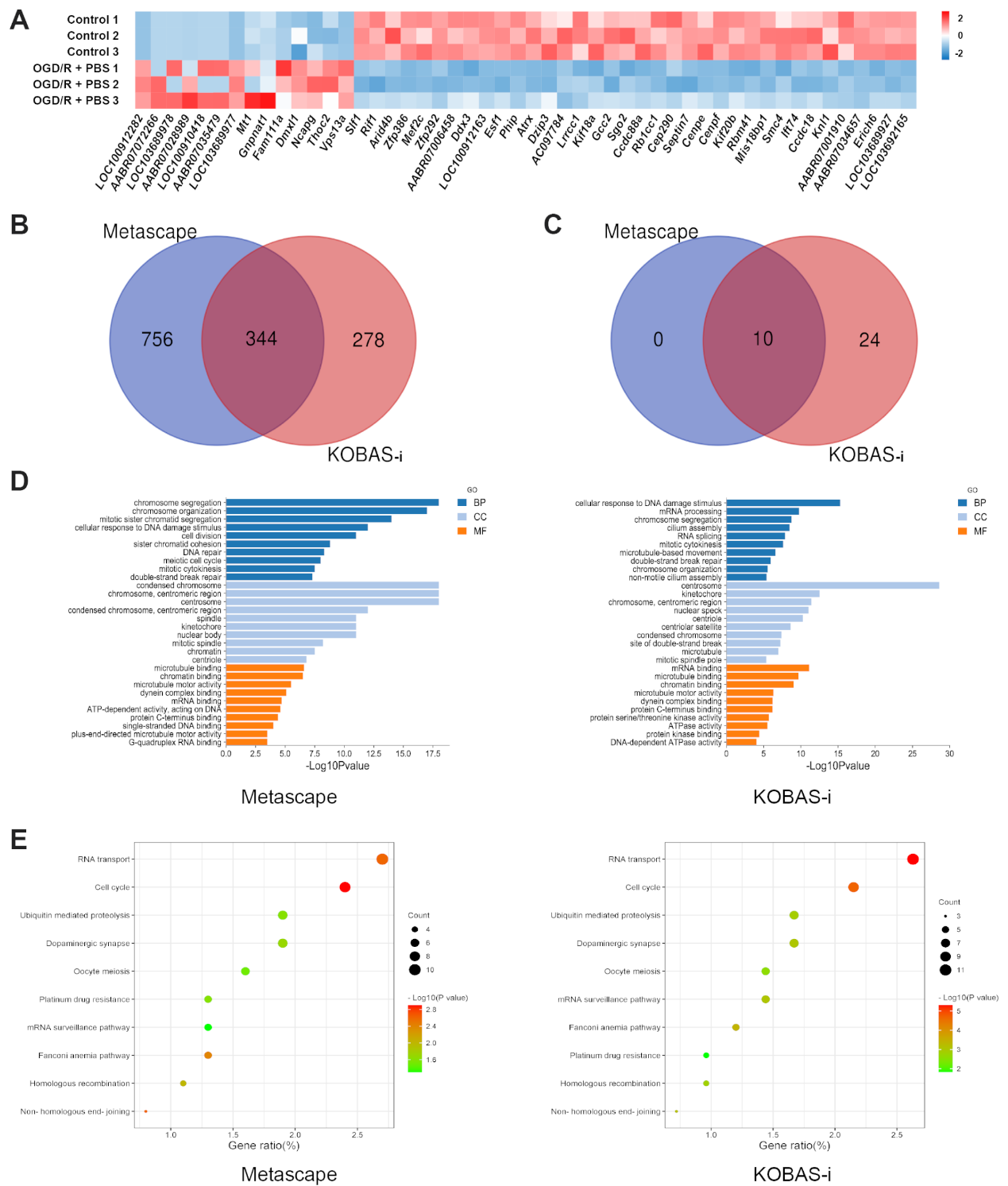


**Fig.S6 Differential expression between control and OGD/R + PBS groups. (A)** Top 50 DEGs between control and OGD/R + PBS groups, venn diagram of the intersection of DEGs enrichment analysis results respectively performed by **(B)** GO and **(C)** KEGG enrichment analysis based on both Metascape and KOBAS-I; **(D)** top 10 enriched GO terms in BP, CC and MF sections respectively using Metascape and KOBAS-I; **(E)** KEGG pathway analysis respectively using Metascape and KOBAS-i: the enriched significance gradually increases from green to red, and the dot sizes indicate the number of DEGs contained in the corresponding pathway.


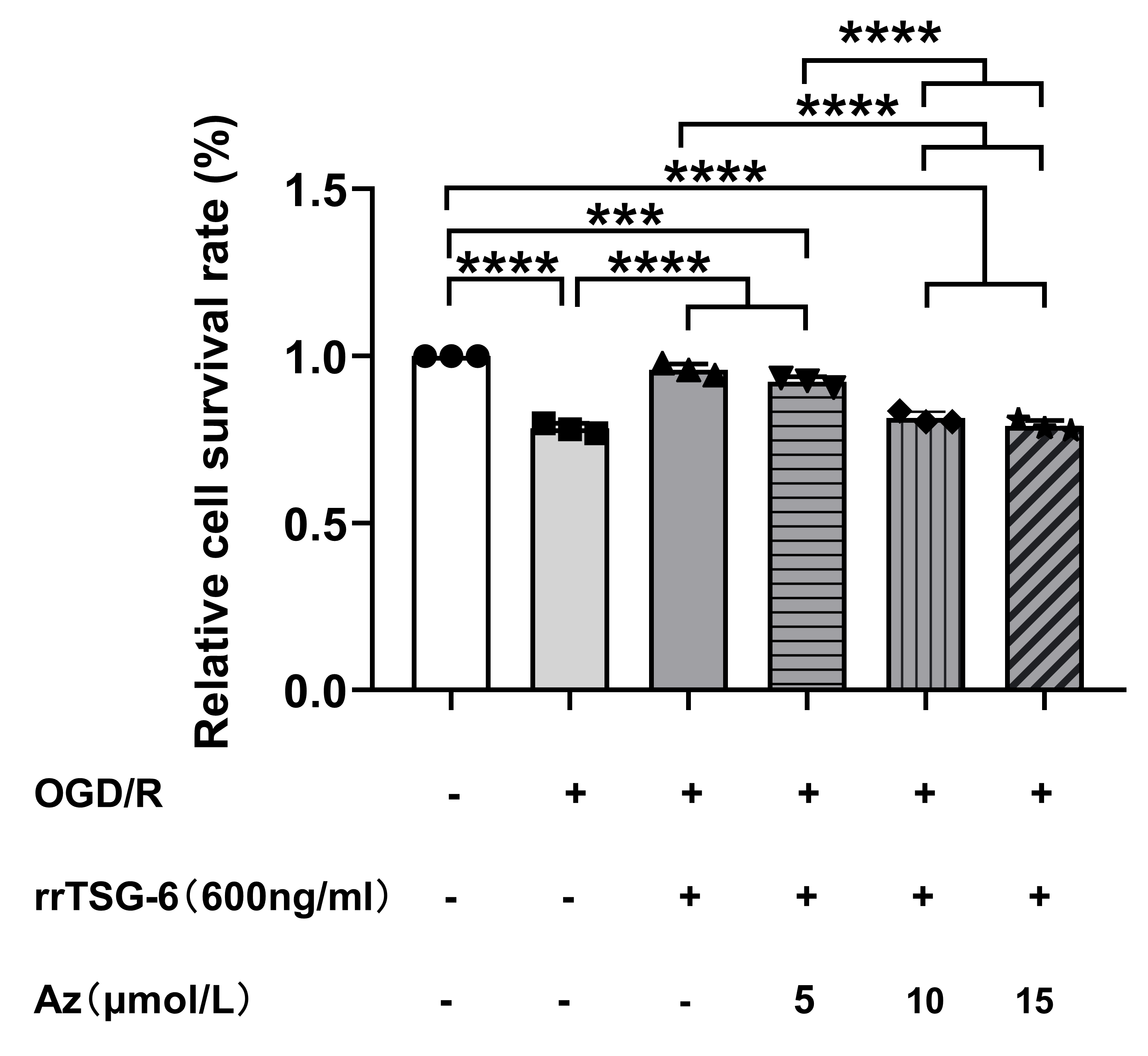


**Fig.S7 Astrocyte viability at 24 h following OGD/R**. Astrocyte viability was determined by MTS assay at 24 h following OGD/R. ^***^*P* < 0.01, ^****^*P* < 0.001, all values are showed as mean ± standard deviation, one-way ANOVA, *n* = 3.

**~~Table.S1 Clean reads data~~**

| **Sample** | **Clean Reads** | **Clean Bases（G）** | **Q20%** | **Q30%** | **GC content%** |
| --- | --- | --- | --- | --- | --- |
| Control 1 | 45226510 | 6.78G | 99.95 | 98.72 | 50.50 |
| Control 2 | 39798004 | 5.97G | 99.98 | 98.87 | 50.50 |
| Control 3 | 51080466 | 7.66G | 99.94 | 98.35 | 50.00 |
| OGD/R + PBS 1 | 41844658 | 6.28G | 99.93 | 98.22 | 51.00 |
| OGD/R + PBS 2 | 39121510 | 5.87G | 99.95 | 98.39 | 51.00 |
| OGD/R + PBS 3 | 49867586 | 7.48G | 99.93 | 98.19 | 50.50 |
| OGD/R + rrTSG-6 1 | 52553840 | 7.88G | 99.94 | 98.26 | 50.00 |
| OGD/R + rrTSG-6 2 | 52110828 | 7.82G | 99.93 | 98.26 | 51.00 |
| OGD/R + rrTSG-6 3 | 50938804 | 7.64G | 99.94 | 98.36 | 50.00 |

**Table.S2 Overall comparison between reads and reference genome sequences (1)**

| **Sample** | **Total reads** | **Total Mapped** | **Unique Mapped** | **Multi Mapped** | **PE Mapped** |
| --- | --- | --- | --- | --- | --- |
| Control 1 | 45226510 | 38452500  (85.02%) | 29773669  (65.83%) | 8678831  (19.19%) | 36436030  (80.56%) |
| Control 2 | 39798004 | 34101255  (85.69%) | 26328221  (66.15%) | 7773034  (19.53%) | 32323294  (81.22%) |
| Control 3 | 51080466 | 43205636  (84.58%) | 33282611  (65.16%) | 9923025  (19.43%) | 40898732  (80.07%) |
| OGD/R + PBS 1 | 41844658 | 35137439  (83.97%) | 26967505  (64.45%) | 8169934  (19.52%) | 33274326  (79.52%) |
| OGD/R + PBS 2 | 39121510 | 32877751  (84.04%) | 25281633  (64.62%) | 7596118  (19.42%) | 31146450  (79.61%) |
| OGD/R + PBS 3 | 49867586 | 42060996  (84.35%) | 32181204  (64.53%) | 9879792  (19.81%) | 39820742  (79.85%) |
| OGD/R + rrTSG-6 1 | 52553840 | 44783077  (85.21%) | 34433988  (65.52%) | 10349089  (19.69%) | 42201196  (80.30%) |
| OGD/R + rrTSG-6 2 | 52110828 | 43971691  (84.38%) | 33676758  (64.63%) | 10294933  (19.76%) | 41627926  (79.88%) |
| OGD/R + rrTSG-6 3 | 50938804 | 43434333  (85.27%) | 33517840  (65.80%) | 9916493  (19.47%) | 41068168  (80.62%) |

**Table.S3 Overall comparison between reads and reference genome sequences (2)**

| **Sample** | **Reads map to sense strand** | **Reads map to antisense strand** | **Non-splice** | **Splice reads** |
| --- | --- | --- | --- | --- |
| Control 1 | 18157823  (40.15%) | 18171027  (40.18%) | 21152172  (46.77%) | 15176678  (33.56%) |
| Control 2 | 16070344  (40.38%) | 16086809  (40.42%) | 18868463  (47.41%) | 13288690  (33.39%) |
| Control 3 | 20396761  (39.93%) | 20416523  (39.97%) | 23983183  (46.95%) | 16830101  (32.95%) |
| OGD/R + PBS 1 | 16606642  (39.69%) | 16619941  (39.72%) | 18910319  (45.19%) | 14316264  (34.21%) |
| OGD/R + PBS 2 | 15545539  (39.74%) | 15558518  (39.77%) | 17450466  (44.61%) | 13653591  (34.90%) |
| OGD/R + PBS 3 | 19844044  (39.79%) | 19862117  (39.83%) | 22762813  (45.65%) | 16943348  (33.98%) |
| OGD/R +rrTSG-6 1 | 21177636  (40.30%) | 21197697  (40.34%) | 24843119  (47.27%) | 17532214  (33.36%) |
| OGD/R + rrTSG-6 2 | 20753771  (39.83%) | 20771162  (39.86%) | 23551959  (45.20%) | 17972974  (34.49%) |
| OGD/R + rrTSG-6 3 | 20521214  (40.29%) | 20537338  (40.32%) | 24028790  (47.17%) | 17029762  (33.43%) |

**Table.S4 Primer sequences**

|  | **Forward (5’-3’)** | **Reverse (5’-3’)** |
| --- | --- | --- |
| ***Tnfaip6*** | AGGTGGCCGTCTTGCAACCT | ATGCCGGTTTTGCCAAATCC |
| ***Tnf-α*** | TCAAGAGCCCTTGCCCTAAG | TGGAAGACTCCTCCCAGGTA |
| ***Il-6*** | CCACTGCCTTCCCTACTTCA | ACAGTGCATCATCGCTGTTC |
| ***Il-1β*** | GGGATGATGACGACCTGCTA | TGTCGTTGCTTGTCTCTCCT |
| ***Hspa1b*** | CGTGGAGGAGTTCAAGAGGA | GCGTGATGGACGTGTAGAAG |
| ***β-Actin*** | TGGCCCCTGAGGAGCACCCT | TGGATGGCTACGTACATGGC |

**Table.S5 351 differentially expressed genes (DEGs)** between A and B groups

| Gene_name | logFC | P.Value |
| --- | --- | --- |
| Kcnj13 | -1.733079763 | 0.000331065 |
| Clic6 | -1.590095715 | 0.024421102 |
| Islr | -1.556091446 | 0.03048929 |
| 1190005I06Rik | -1.471099176 | 0.007278356 |
| Folr1 | -1.398138291 | 0.044880181 |
| Cml5 | -1.362506702 | 0.000397549 |
| Ifit2 | -1.352383663 | 0.017383379 |
| Gm5089 | -1.284442574 | 0.001679374 |
| 2610034M16Rik | -1.249619747 | 0.000443622 |
| Gpx3 | -1.237273744 | 0.000681538 |
| Hmgcs2 | -1.228247321 | 0.012842813 |
| Prelp | -1.208995068 | 0.009199945 |
| Gdf10 | -1.157605332 | 0.002523053 |
| C3 | -1.143992832 | 0.019757736 |
| Pacrg | -1.14007467 | 0.010396145 |
| Pdk2 | -1.119777531 | 0.008948197 |
| Gpr137b | -1.08966874 | 0.004213117 |
| Cmbl | -1.072420602 | 0.004070462 |
| Rdh5 | -1.071490842 | 0.007661528 |
| Hnmt | -1.066606926 | 0.003791026 |
| Padi2 | -1.06479063 | 0.011097779 |
| Fam227b | -1.060848846 | 0.018281464 |
| Ephx1 | -1.054492283 | 0.002012201 |
| Aldh1a1 | -1.043033845 | 0.002644918 |
| Gsta4 | -1.030598461 | 0.002989665 |
| Ptgr1 | -1.010771009 | 0.034240071 |
| Gsta2 | -1.009576672 | 0.022658189 |
| Gpr137b-ps | -0.97891253 | 0.0056578 |
| Tmem218 | -0.971881892 | 0.008916301 |
| Nadk2 | -0.965375794 | 0.002418943 |
| Eda2r | -0.944619598 | 0.041909774 |
| Pantr1 | -0.942088506 | 0.007267109 |
| Rbl2 | -0.938673895 | 0.006267513 |
| Kcnk2 | -0.934742446 | 0.022551464 |
| Lrrc75b | -0.930386574 | 0.02712282 |
| Etnppl | -0.924190483 | 0.017164876 |
| Slc37a4 | -0.915710398 | 0.023186163 |
| G0s2 | -0.908582507 | 0.041406699 |
| Selenbp1 | -0.900507919 | 0.001001602 |
| Pdgfd | -0.879027834 | 0.01982977 |
| 1700019G17Rik | -0.872234484 | 0.000760046 |
| Fam47e | -0.863807063 | 0.029416025 |
| Hapln1 | -0.863161982 | 0.000745183 |
| Ranbp3l | -0.862940082 | 0.022327443 |
| Nudt12 | -0.85676613 | 0.009626583 |
| Pik3ip1 | -0.852345647 | 0.024978684 |
| Dbp | -0.841406901 | 0.017518435 |
| Hlf | -0.840593765 | 0.000121834 |
| Blvrb | -0.840513275 | 0.007224624 |
| Mrpl41 | -0.814067414 | 0.003625277 |
| Gbe1 | -0.800451107 | 0.03809644 |
| Hist3h2a | -0.79541146 | 0.01224585 |
| Id3 | -0.791715447 | 0.02629849 |
| Nat8 | -0.786299128 | 0.00159995 |
| Gdpd2 | -0.78622419 | 0.005734801 |
| Adcy8 | -0.778118908 | 0.038276673 |
| Syt14 | -0.766321943 | 0.036553652 |
| Dhtkd1 | -0.761720554 | 0.00418384 |
| Folh1 | -0.754455467 | 0.009856397 |
| Rsph10b | -0.75397885 | 0.001648625 |
| Galm | -0.742171438 | 0.007398895 |
| Lpar4 | -0.74136752 | 0.002083194 |
| Ccng1 | -0.736028381 | 0.017213681 |
| 4933431E20Rik | -0.736004974 | 0.035058376 |
| Id4 | -0.735943272 | 0.001048022 |
| Dpyd | -0.733244496 | 0.000791234 |
| Slc2a12 | -0.729670914 | 0.00255387 |
| Pnpla7 | -0.726409833 | 0.007477313 |
| Aass | -0.720688829 | 0.002949554 |
| Itih5 | -0.718709733 | 0.04959403 |
| Ripk4 | -0.717985111 | 0.047106706 |
| H2afv | -0.717923421 | 0.000836713 |
| Gm11627 | -0.716860763 | 0.000137528 |
| Sostdc1 | -0.716408393 | 0.008338956 |
| Slc13a4 | -0.714062755 | 0.006549574 |
| Slc24a4 | -0.712179571 | 0.003632731 |
| Dnajc9 | -0.710798833 | 0.014792545 |
| Mxi1 | -0.710084296 | 0.01744111 |
| Prom1 | -0.706697974 | 0.031883925 |
| Hacl1 | -0.705189449 | 0.008414597 |
| Hist1h1c | -0.70431426 | 0.003188637 |
| Rbbp9 | -0.704097707 | 0.034447173 |
| Lhx2 | -0.701994759 | 0.000174284 |
| Spag1 | -0.696596991 | 0.016695931 |
| Mettl7a1 | -0.693457886 | 0.006407101 |
| Decr1 | -0.689167372 | 0.006229551 |
| Pcmtd2 | -0.688991365 | 0.031379025 |
| Efhd1 | -0.688156732 | 0.025786783 |
| Chdh | -0.68767253 | 0.013177972 |
| Gclm | -0.686285355 | 0.030364746 |
| Clybl | -0.685444728 | 0.047533931 |
| Trim12c | -0.684071998 | 0.042046489 |
| Nqo1 | -0.675759479 | 0.042691772 |
| Mcm7 | -0.673019456 | 0.023796441 |
| 1700007K13Rik | -0.672670519 | 0.030076397 |
| B230354K17Rik | -0.663971657 | 0.030494287 |
| Tcfl5 | -0.663193699 | 0.006627095 |
| Cldn10 | -0.662622894 | 0.004403842 |
| Gm9925 | -0.660771093 | 0.026979224 |
| Acad11 | -0.658691641 | 0.000784413 |
| Pamr1 | -0.657784399 | 0.036375439 |
| Fbxo2 | -0.655430916 | 0.000166358 |
| Atp5s | -0.647321426 | 0.000503049 |
| Msrb1 | -0.644178168 | 0.006274143 |
| Nkain4 | -0.641999623 | 0.003980256 |
| Tprkb | -0.641086095 | 0.006727366 |
| Dync2li1 | -0.638753334 | 0.034500906 |
| Exd1 | -0.638681553 | 0.001673338 |
| Fcgrt | -0.636738524 | 0.013053682 |
| Ccpg1 | -0.636569621 | 0.025180379 |
| Kiz | -0.634632834 | 0.014261749 |
| Katnal2 | -0.633657474 | 0.00119474 |
| l7Rn6 | -0.633090567 | 0.024220036 |
| Bdh2 | -0.632696804 | 0.019331429 |
| Pfn4 | -0.63140091 | 0.027018191 |
| Gcdh | -0.631051745 | 0.04417801 |
| Gm5083 | -0.629067362 | 0.026433592 |
| Slc37a2 | -0.628242971 | 0.003729694 |
| Sulf2 | -0.627560157 | 0.002674914 |
| Ttc38 | -0.626773876 | 0.002576607 |
| Zfp946 | -0.625324164 | 0.028244944 |
| Tldc1 | -0.624918536 | 0.001010863 |
| Ap3m1 | -0.624403665 | 0.006950907 |
| Fabp7 | -0.622695092 | 0.004173126 |
| Gm17750 | -0.620364498 | 0.008212942 |
| Klhdc8b | -0.61946621 | 0.000879777 |
| Cldn2 | -0.619163699 | 0.016116541 |
| Fundc1 | -0.618844827 | 0.011685649 |
| Ccdc122 | -0.617742809 | 0.000794517 |
| Pla2g16 | -0.616949532 | 0.018478507 |
| Slc35a5 | -0.616131569 | 0.007110359 |
| Rsad2 | -0.61549826 | 0.000716421 |
| 4931406C07Rik | -0.614959432 | 0.003695862 |
| Lix1 | -0.614277563 | 0.01698377 |
| Taldo1 | -0.613750987 | 0.006977516 |
| Fam229b | -0.613418335 | 0.007472214 |
| Tmem27 | -0.611747333 | 0.008862645 |
| Sesn3 | -0.611325659 | 0.010187948 |
| Pygl | -0.611085401 | 0.014510953 |
| Gm2115 | -0.609756465 | 0.022001394 |
| Csrp1 | -0.607988993 | 0.029005516 |
| Aamdc | -0.607251582 | 0.023442049 |
| Sult1a1 | -0.606601918 | 0.004043444 |
| Gm11681 | -0.605107864 | 0.013869574 |
| Tcp11l2 | -0.602143584 | 0.003846899 |
| Slc48a1 | -0.601534982 | 0.000934368 |
| Asrgl1 | -0.601297578 | 0.00394922 |
| Pkia | -0.600127588 | 0.046842761 |
| Trim30a | -0.59991616 | 0.043737293 |
| Hrsp12 | -0.59820252 | 0.006124494 |
| Bmyc | -0.597380486 | 0.010612533 |
| Ccbl2 | -0.59507227 | 0.036890877 |
| Hspa2 | -0.592411682 | 0.018485248 |
| Cript | -0.590990264 | 0.002375372 |
| Tmem53 | -0.589825317 | 0.018509561 |
| Adhfe1 | -0.588663422 | 0.019957196 |
| 4933416E14Rik | -0.584145391 | 0.004935376 |
| Klhdc1 | -0.582178893 | 0.049241219 |
| Acaa2 | -0.581994381 | 0.005421058 |
| Abcc4 | -0.581615912 | 0.008106083 |
| Rps6ka6 | -0.579518489 | 0.043970935 |
| Txndc16 | -0.578336829 | 0.03700436 |
| Nudt1 | -0.577160848 | 0.043266515 |
| Abca8a | -0.576572726 | 0.008905113 |
| D930002L09Rik | -0.576391546 | 0.024195478 |
| Cep128 | -0.575952841 | 0.034968386 |
| Gprc5b | -0.572107023 | 0.000768797 |
| Hsd17b11 | -0.571933294 | 0.02851227 |
| Ppm1k | -0.571212418 | 0.009063713 |
| 9230114K14Rik | -0.570761065 | 0.03215757 |
| Spidr | -0.566828549 | 0.004056489 |
| Krcc1 | -0.563631475 | 0.027230924 |
| Pgd | -0.562481628 | 0.019338575 |
| Tcf24 | -0.561345547 | 0.021829841 |
| Acot13 | -0.558966621 | 0.011683253 |
| Gstz1 | -0.558324884 | 0.016814268 |
| Dcxr | -0.556916121 | 0.025885865 |
| Olfml1 | -0.556637817 | 0.031492931 |
| Pla2g3 | -0.555791431 | 0.040430375 |
| Cd1d1 | -0.554989141 | 0.042613056 |
| Commd9 | -0.554235204 | 0.00078819 |
| Elmod3 | -0.553561886 | 0.036270003 |
| Mr1 | -0.552162251 | 0.015899075 |
| Lmcd1 | -0.549770885 | 0.009002651 |
| Entpd5 | -0.548525943 | 0.011780413 |
| Cyb5a | -0.548341941 | 0.023082799 |
| Ddo | -0.548086436 | 0.005345107 |
| Arhgef10 | -0.547504589 | 0.025488313 |
| Iyd | -0.54741504 | 0.044591286 |
| Mettl20 | -0.547212031 | 0.033304626 |
| Hsdl2 | -0.54717111 | 0.048376441 |
| Rpa3 | -0.546721284 | 0.013071936 |
| Fmo1 | -0.546076067 | 0.018314718 |
| Gsto2 | -0.545280432 | 0.000264748 |
| Pxmp2 | -0.54493838 | 0.000600167 |
| Myom3 | -0.544892602 | 0.032122815 |
| Cryab | -0.544714824 | 0.022760486 |
| Cpxm1 | -0.542923087 | 0.014949798 |
| Fars2 | -0.541028841 | 0.03612059 |
| Fam189a2 | -0.53599623 | 0.002040555 |
| Klhl24 | -0.53516709 | 0.040631776 |
| Spata1 | -0.534515999 | 0.034682385 |
| Morn2 | -0.534031205 | 0.033858558 |
| Sat2 | -0.532513652 | 0.03223383 |
| C130030K03Rik | -0.532160477 | 0.049343569 |
| Plscr4 | -0.530516456 | 0.038008259 |
| Rnf182 | -0.530360491 | 0.008222011 |
| Dctn3 | -0.529492043 | 0.04874921 |
| Grhpr | -0.525379445 | 0.023543836 |
| Magi2 | -0.525360912 | 0.03092609 |
| Kcnn1 | -0.525029871 | 0.002297972 |
| Cat | -0.524628758 | 0.031658121 |
| BC064078 | -0.523754668 | 0.018180149 |
| Ipp | -0.522967348 | 0.019264386 |
| Cml1 | -0.521727941 | 0.00754417 |
| Asl | -0.521272328 | 0.038831314 |
| Ralgps2 | -0.51872763 | 0.001674963 |
| Ubxn8 | -0.518675248 | 0.028336709 |
| Abhd12 | -0.517911448 | 0.024037981 |
| Dhrs3 | -0.517148234 | 0.009050084 |
| Retsat | -0.516723748 | 0.014751551 |
| Calhm2 | -0.515516216 | 0.027030555 |
| Mmd | -0.514791705 | 0.034592195 |
| Abhd1 | -0.511101995 | 0.017754716 |
| Zfyve21 | -0.510749926 | 0.049330292 |
| Aldh3a2 | -0.508252376 | 0.014718627 |
| Pck2 | -0.507971242 | 0.000453337 |
| Plgrkt | -0.507690418 | 0.036679066 |
| Ift46 | -0.507577597 | 0.031773672 |
| Dars2 | -0.507566918 | 0.044212346 |
| Hibch | -0.507468794 | 0.009919366 |
| Nde1 | -0.507253659 | 0.045315862 |
| Phyhipl | -0.506470464 | 0.015997644 |
| Nrarp | -0.505126047 | 0.023752372 |
| Klhl8 | -0.503362575 | 0.025317626 |
| Cbr4 | -0.503336611 | 0.008555339 |
| Lhpp | -0.502044437 | 0.011585777 |
| Cotl1 | -0.501802605 | 0.013034357 |
| Efcab7 | -0.50154095 | 0.003498961 |
| Pcbd2 | -0.500784017 | 0.037693233 |
| Srl | -0.500242271 | 0.03971498 |
| Pabpn1 | 0.501251403 | 0.001853552 |
| 2600002B07Rik | 0.501680227 | 0.046489154 |
| Sipa1 | 0.503121298 | 0.020778224 |
| Ncald | 0.503987356 | 0.005659133 |
| Tnc | 0.50408812 | 0.034589575 |
| Rabggta | 0.506573085 | 0.002763391 |
| Flrt3 | 0.511900502 | 0.010476063 |
| Cd44 | 0.514217786 | 0.007442831 |
| Slc6a17 | 0.515630832 | 0.030605867 |
| Trrap | 0.519387817 | 0.036814744 |
| Arc | 0.519875515 | 0.002848256 |
| Synpo | 0.520256841 | 0.008263468 |
| Vgf | 0.523323407 | 0.034104619 |
| Nedd9 | 0.527762223 | 0.047566939 |
| Ptgfrn | 0.528214566 | 0.020021631 |
| Chd7 | 0.529080477 | 0.043630064 |
| Pxdn | 0.533444451 | 0.022294364 |
| Loxl1 | 0.53551979 | 0.002458784 |
| Gpr176 | 0.537994953 | 0.007407471 |
| C330006D17Rik | 0.539995246 | 0.033949088 |
| D9Ertd256e | 0.541307368 | 0.020114376 |
| Flna | 0.544208777 | 0.001816947 |
| Ckap4 | 0.545224157 | 0.021608492 |
| Shisa6 | 0.545345487 | 0.018033783 |
| Map4k3 | 0.546180226 | 0.005389213 |
| Gm10796 | 0.548601202 | 0.020305287 |
| Gpr3 | 0.549098997 | 0.030763401 |
| Fbn2 | 0.551198053 | 0.041016909 |
| Camk1d | 0.553086646 | 0.030070308 |
| Trim47 | 0.560026479 | 0.002969745 |
| Adamtsl2 | 0.570141701 | 0.003491089 |
| Tor4a | 0.573220434 | 0.00199419 |
| Cd14 | 0.573497909 | 0.03089816 |
| 2810427A07Rik | 0.574102851 | 0.024559747 |
| Cthrc1 | 0.581392042 | 0.022357439 |
| U2af1 | 0.592989804 | 0.024730558 |
| Maff | 0.596888402 | 0.046414237 |
| Mrpl18 | 0.597554272 | 0.035880764 |
| Mtmr11 | 0.600955307 | 0.026559922 |
| C78505 | 0.605658724 | 0.027859477 |
| Mir22hg | 0.607012128 | 0.015680284 |
| Ezh2 | 0.610845922 | 0.001789524 |
| C230066G23Rik | 0.620086956 | 0.038922865 |
| Rhob | 0.624122985 | 0.00118959 |
| Sema4c | 0.624708948 | 0.001320765 |
| C730049O14Rik | 0.625788838 | 0.000974965 |
| Dennd3 | 0.626870758 | 0.014042157 |
| Pus7 | 0.627447426 | 0.028739958 |
| C76876 | 0.634949853 | 0.025511413 |
| Iqgap2 | 0.635717688 | 0.024570312 |
| Fblim1 | 0.63877539 | 0.016906752 |
| Insig1 | 0.644207711 | 0.000402984 |
| Adam12 | 0.6484854 | 0.046021322 |
| 9030419F21Rik | 0.648904996 | 0.029692112 |
| Gm3924 | 0.654184851 | 0.026269501 |
| Tac2 | 0.659761899 | 0.021955135 |
| Mogs | 0.669564598 | 0.006721984 |
| Eif2ak4 | 0.676583409 | 0.029618542 |
| Nop56 | 0.681931763 | 0.026023825 |
| Rhox4b | 0.685189611 | 0.005288194 |
| Slc7a5 | 0.685761521 | 0.040345939 |
| Rnf125 | 0.692543845 | 0.003466544 |
| Ankrd52 | 0.69338199 | 0.001986378 |
| Plcxd2 | 0.695296245 | 0.00776611 |
| Prg4 | 0.697071322 | 0.005422372 |
| 9030607L20Rik | 0.699949294 | 0.034727205 |
| Kdm6b | 0.705845368 | 0.027656587 |
| Pde2a | 0.706051426 | 0.011047588 |
| Pcdh19 | 0.706171175 | 0.03588873 |
| Phox2a | 0.70756804 | 0.020987342 |
| Ndel1 | 0.712361552 | 0.046612076 |
| Snhg4 | 0.713133687 | 0.001993721 |
| Synm | 0.713214556 | 0.019871805 |
| Rps25 | 0.715517845 | 0.00783018 |
| Adamts9 | 0.717566728 | 0.023327987 |
| Slc5a3 | 0.718906857 | 0.002679762 |
| Socs3 | 0.719501551 | 0.028599069 |
| C80425 | 0.719810332 | 0.032189826 |
| Vash1 | 0.725542659 | 0.027577935 |
| Adamts1 | 0.730691676 | 0.007076759 |
| Rhbdf1 | 0.754106477 | 0.006330783 |
| 2310079F09Rik | 0.75478362 | 0.027462848 |
| Slc4a7 | 0.762597469 | 0.046800544 |
| Dhcr7 | 0.762824852 | 0.024651635 |
| Crispld2 | 0.771590192 | 0.03570608 |
| Nfkbiz | 0.804569242 | 0.005419087 |
| Cxcl1 | 0.815722737 | 0.003546435 |
| Endod1 | 0.832880806 | 0.003301664 |
| A030007N12Rik | 0.842306471 | 0.016386389 |
| Frat2 | 0.84714689 | 0.001217926 |
| Tgm1 | 0.866351024 | 0.032609294 |
| Trib1 | 0.872779766 | 0.019814147 |
| Timp1 | 0.874232005 | 0.023117465 |
| Crlf1 | 0.888035943 | 0.045675928 |
| C77534 | 0.913545487 | 0.010260733 |
| Cxcl3 | 0.923841632 | 0.00701131 |
| Ptprn | 0.976085362 | 0.00732643 |
| Rhoj | 1.00821387 | 0.031121913 |
| Gm1673 | 1.038623578 | 0.006722436 |
| Thbd | 1.079822615 | 0.003976502 |
| AW555355 | 1.11832342 | 0.047904416 |
| Basp1 | 1.146228371 | 0.036865773 |
| Cxcl2 | 1.16457457 | 0.04084905 |
| Spp1 | 1.251036046 | 0.033025638 |
| Bdnf | 1.266897426 | 0.018168127 |
| Cdc42ep3 | 1.270399807 | 0.012695151 |
| Il11 | 1.279732961 | 0.003263239 |
| BC023969 | 1.304478182 | 0.028350442 |
| AI506816 | 1.634515484 | 0.006110189 |
| Il6 | 1.891755398 | 0.03024969 |

**Table.S6 418 DEGs between control and OGD/R + PBS groups.**

| gene_id | gene_name |
| --- | --- |
| ENSRNOG00000048982 | LOC100912282 |
| ENSRNOG00000029216 | AABR07072266 |
| ENSRNOG00000048136 | LOC103689978 |
| ENSRNOG00000050441 | AABR07028989 |
| ENSRNOG00000060898 | LOC100910418 |
| ENSRNOG00000022599 | AABR07035479 |
| ENSRNOG00000052613 | LOC103689977 |
| ENSRNOG00000054614 | AABR07002779 |
| ENSRNOG00000061438 | LOC100360491 |
| ENSRNOG00000011292 | NEWGENE_621351 |
| ENSRNOG00000045554 | LOC102551901 |
| ENSRNOG00000038047 | Mt1 |
| ENSRNOG00000031381 | AC129049 |
| ENSRNOG00000008641 | Gnpnat1 |
| ENSRNOG00000050419 | Avil |
| ENSRNOG00000011151 | Tenm4 |
| ENSRNOG00000015455 | Spr |
| ENSRNOG00000028543 | AABR07052523 |
| ENSRNOG00000018911 | Pfkfb3 |
| ENSRNOG00000049097 | Rpl7a |
| ENSRNOG00000014117 | Hmox1 |
| ENSRNOG00000001274 | Vps29 |
| ENSRNOG00000033152 | Rps18l1 |
| ENSRNOG00000051624 | Hspe1 |
| ENSRNOG00000012099 | Tent2 |
| ENSRNOG00000024213 | Golim4 |
| ENSRNOG00000004415 | Togaram1 |
| ENSRNOG00000009248 | Pnrc2 |
| ENSRNOG00000018946 | Trim33 |
| ENSRNOG00000011024 | Zdhhc20 |
| ENSRNOG00000012414 | Rhobtb3 |
| ENSRNOG00000005234 | Gxylt1 |
| ENSRNOG00000016067 | Ckap5 |
| ENSRNOG00000001597 | Atf2 |
| ENSRNOG00000006792 | Cep57 |
| ENSRNOG00000012500 | Ddx10 |
| ENSRNOG00000004925 | Ppp1r12a |
| ENSRNOG00000007175 | Mier1 |
| ENSRNOG00000012641 | Sfr1 |
| ENSRNOG00000000648 | Jmjd1c |
| ENSRNOG00000042309 | Kiaa0408L |
| ENSRNOG00000005713 | Ccdc82 |
| ENSRNOG00000011417 | Pde3b |
| ENSRNOG00000001682 | Ttc3 |
| ENSRNOG00000005932 | Megf9 |
| ENSRNOG00000014080 | Kif23 |
| ENSRNOG00000025028 | Prkdc |
| ENSRNOG00000001616 | Senp7 |
| ENSRNOG00000000415 | Asf1a |
| ENSRNOG00000020701 | Brca1 |
| ENSRNOG00000024336 | Senp6 |
| ENSRNOG00000038035 | Kif4a |
| ENSRNOG00000036604 | Ifit2 |
| ENSRNOG00000011756 | Phf3 |
| ENSRNOG00000018149 | Smarca5 |
| ENSRNOG00000028930 | Dab2 |
| ENSRNOG00000002871 | Rbm25l1 |
| ENSRNOG00000013055 | Zfyve16 |
| ENSRNOG00000017447 | Eif2s2 |
| ENSRNOG00000055012 | LOC108348078 |
| ENSRNOG00000006864 | Scaper |
| ENSRNOG00000032070 | Dync2h1 |
| ENSRNOG00000031053 | Mt-nd4l |
| ENSRNOG00000016690 | Idi1 |
| ENSRNOG00000053635 | Zkscan8 |
| ENSRNOG00000016252 | Mblac2 |
| ENSRNOG00000017791 | Arhgap12 |
| ENSRNOG00000028358 | Edem3 |
| ENSRNOG00000012806 | Rbbp6 |
| ENSRNOG00000006329 | Peli1 |
| ENSRNOG00000005927 | Cpsf6 |
| ENSRNOG00000016266 | Mphosph10 |
| ENSRNOG00000024998 | Trps1 |
| ENSRNOG00000056246 | Gls |
| ENSRNOG00000053047 | Top2a |
| ENSRNOG00000059714 | Hsp90aa1 |
| ENSRNOG00000016377 | Cep55 |
| ENSRNOG00000018322 | Picalm |
| ENSRNOG00000010515 | Trim59 |
| ENSRNOG00000051680 | Ccdc186 |
| ENSRNOG00000019848 | Rbm39 |
| ENSRNOG00000013798 | Fnbp1l |
| ENSRNOG00000007682 | Gria3 |
| ENSRNOG00000024650 | Ckap2 |
| ENSRNOG00000028227 | Pbrm1 |
| ENSRNOG00000010027 | Atr |
| ENSRNOG00000012881 | Fgl2 |
| ENSRNOG00000024194 | Rsf1 |
| ENSRNOG00000013078 | Zcchc7 |
| ENSRNOG00000004127 | Cep170 |
| ENSRNOG00000048922 | LOC100909664 |
| ENSRNOG00000001813 | Dnm1l |
| ENSRNOG00000006001 | Luc7l2 |
| ENSRNOG00000006412 | Zhx1 |
| ENSRNOG00000029055 | Ttk |
| ENSRNOG00000016338 | Fam92a |
| ENSRNOG00000004186 | Snx13 |
| ENSRNOG00000024863 | Fam76b |
| ENSRNOG00000025025 | Dnttip2 |
| ENSRNOG00000022499 | Sgo1 |
| ENSRNOG00000001098 | Pds5b |
| ENSRNOG00000024365 | Ect2 |
| ENSRNOG00000003901 | Cfap36 |
| ENSRNOG00000003559 | Ccdc112 |
| ENSRNOG00000046366 | Dmd |
| ENSRNOG00000057806 | Trpm7 |
| ENSRNOG00000009104 | AABR07029605 |
| ENSRNOG00000011245 | Arhgap18 |
| ENSRNOG00000005258 | Myef2 |
| ENSRNOG00000029185 | Rasa1 |
| ENSRNOG00000006980 | Vcpip1 |
| ENSRNOG00000009795 | Nfib |
| ENSRNOG00000010286 | Cast |
| ENSRNOG00000014844 | Kif21a |
| ENSRNOG00000004861 | Itga4 |
| ENSRNOG00000007719 | Ccnc |
| ENSRNOG00000005730 | Pcmtd1 |
| ENSRNOG00000058663 | Suz12 |
| ENSRNOG00000021718 | Cspp1 |
| ENSRNOG00000046621 | AABR07043748 |
| ENSRNOG00000002585 | Cul4b |
| ENSRNOG00000022015 | Cntrl |
| ENSRNOG00000019180 | Acsl4 |
| ENSRNOG00000014599 | Dhx36 |
| ENSRNOG00000008039 | Cul5 |
| ENSRNOG00000054751 | Lmbrd2 |
| ENSRNOG00000003919 | Rps6kb1 |
| ENSRNOG00000007884 | Bcap29 |
| ENSRNOG00000011619 | Myo9a |
| ENSRNOG00000006241 | Marchf7 |
| ENSRNOG00000047611 | Top1 |
| ENSRNOG00000007937 | Krit1 |
| ENSRNOG00000020605 | Pstk |
| ENSRNOG00000010418 | Nek1 |
| ENSRNOG00000025768 | Clk1 |
| ENSRNOG00000012318 | Aspm |
| ENSRNOG00000014320 | Inhba |
| ENSRNOG00000014509 | Sacs |
| ENSRNOG00000020423 | Apc |
| ENSRNOG00000003889 | Tbc1d15 |
| ENSRNOG00000001598 | Usp16 |
| ENSRNOG00000009990 | Zranb2 |
| ENSRNOG00000008629 | Secisbp2l |
| ENSRNOG00000025554 | Zfp445 |
| ENSRNOG00000023593 | Upf2 |
| ENSRNOG00000030314 | Golgb1 |
| ENSRNOG00000000593 | Rev3l |
| ENSRNOG00000038436 | RGD1307100 |
| ENSRNOG00000047620 | Golph3l |
| ENSRNOG00000006378 | Mga |
| ENSRNOG00000008626 | Manea |
| ENSRNOG00000010084 | Ythdf3 |
| ENSRNOG00000011339 | Slk |
| ENSRNOG00000051480 | Fxr1 |
| ENSRNOG00000011654 | Plk4 |
| ENSRNOG00000015334 | Fcho2 |
| ENSRNOG00000022337 | Slitrk6 |
| ENSRNOG00000004677 | Zeb2 |
| ENSRNOG00000011987 | Cd2ap |
| ENSRNOG00000031421 | Eif1a |
| ENSRNOG00000053122 | Scn1a |
| ENSRNOG00000006867 | Etv1 |
| ENSRNOG00000003714 | Clk4 |
| ENSRNOG00000056716 | Zbtb20 |
| ENSRNOG00000004810 | Plcb1 |
| ENSRNOG00000004574 | Ofd1 |
| ENSRNOG00000011317 | Pkn2 |
| ENSRNOG00000010185 | RGD1306941 |
| ENSRNOG00000000397 | Ccar1 |
| ENSRNOG00000060356 | Kif15 |
| ENSRNOG00000009196 | Rc3h2 |
| ENSRNOG00000008393 | Tax1bp1 |
| ENSRNOG00000001989 | Alcam |
| ENSRNOG00000011498 | Psip1 |
| ENSRNOG00000057713 | Cav2 |
| ENSRNOG00000003611 | Dynlt3 |
| ENSRNOG00000007662 | Zfp800 |
| ENSRNOG00000014173 | Smc3 |
| ENSRNOG00000008788 | Mpp5 |
| ENSRNOG00000057464 | Fmr1 |
| ENSRNOG00000017864 | Bdp1 |
| ENSRNOG00000054011 | Tbc1d8b |
| ENSRNOG00000009057 | Sec62 |
| ENSRNOG00000058050 | Bclaf1 |
| ENSRNOG00000013727 | Ndc80 |
| ENSRNOG00000008859 | Tank |
| ENSRNOG00000029342 | Scn7a |
| ENSRNOG00000004232 | G2e3 |
| ENSRNOG00000013491 | Setx |
| ENSRNOG00000005386 | Kitlg |
| ENSRNOG00000047194 | Arl13b |
| ENSRNOG00000028380 | Ccdc66 |
| ENSRNOG00000007804 | C1galt1 |
| ENSRNOG00000028677 | LOC361346 |
| ENSRNOG00000004061 | Pnn |
| ENSRNOG00000022999 | Ppp2r3a |
| ENSRNOG00000012899 | Rbbp8 |
| ENSRNOG00000024964 | Nufip2 |
| ENSRNOG00000010117 | Eif3a |
| ENSRNOG00000048315 | Eif2ak2 |
| ENSRNOG00000003242 | Gulp1 |
| ENSRNOG00000014076 | Mbnl1 |
| ENSRNOG00000001829 | Ube2v2 |
| ENSRNOG00000026962 | Osbpl8 |
| ENSRNOG00000006526 | Sema3c |
| ENSRNOG00000053060 | Zfc3h1 |
| ENSRNOG00000023356 | Eif5b |
| ENSRNOG00000015692 | Taok1 |
| ENSRNOG00000017642 | Acbd5 |
| ENSRNOG00000026226 | Hook1 |
| ENSRNOG00000006749 | Tmtc3 |
| ENSRNOG00000014030 | Synm |
| ENSRNOG00000003720 | Prrx1 |
| ENSRNOG00000008607 | U2surp |
| ENSRNOG00000012405 | Tcf4 |
| ENSRNOG00000054446 | Sltm |
| ENSRNOG00000039091 | Pnpla8 |
| ENSRNOG00000015734 | Ube3a |
| ENSRNOG00000005158 | Slc24a5 |
| ENSRNOG00000021931 | Uba6 |
| ENSRNOG00000032778 | Bub1 |
| ENSRNOG00000006569 | Itgb8 |
| ENSRNOG00000019175 | Ercc6l2 |
| ENSRNOG00000014319 | Smchd1 |
| ENSRNOG00000056753 | Hectd2 |
| ENSRNOG00000018719 | Cir1 |
| ENSRNOG00000023337 | Sema3a |
| ENSRNOG00000001238 | Rsrc2 |
| ENSRNOG00000054474 | Mastl |
| ENSRNOG00000018011 | Tasor2 |
| ENSRNOG00000029212 | Vcan |
| ENSRNOG00000011829 | Rpgrip1l |
| ENSRNOG00000006684 | Zfp317 |
| ENSRNOG00000009836 | Rbm26 |
| ENSRNOG00000013322 | Pola1 |
| ENSRNOG00000012733 | Ankrd12 |
| ENSRNOG00000014745 | AABR07026654 |
| ENSRNOG00000026239 | Arid4a |
| ENSRNOG00000006797 | Zfp280c |
| ENSRNOG00000013756 | Snrnp48 |
| ENSRNOG00000005292 | Trip11 |
| ENSRNOG00000002153 | Cep135 |
| ENSRNOG00000013002 | Gpbp1 |
| ENSRNOG00000045771 | Chl1 |
| ENSRNOG00000010921 | Taf1d |
| ENSRNOG00000026299 | Mysm1 |
| ENSRNOG00000009782 | Efcab7 |
| ENSRNOG00000027770 | Trpm3 |
| ENSRNOG00000017784 | Lrif1 |
| ENSRNOG00000014139 | Sclt1 |
| ENSRNOG00000039994 | Upf3b |
| ENSRNOG00000014750 | Tasor |
| ENSRNOG00000024934 | Npat |
| ENSRNOG00000009116 | Itgb3bp |
| ENSRNOG00000039740 | Cenpk |
| ENSRNOG00000052062 | Tut4 |
| ENSRNOG00000029220 | Zmym5 |
| ENSRNOG00000012255 | Ktn1 |
| ENSRNOG00000005904 | Cdc27 |
| ENSRNOG00000000161 | Chm |
| ENSRNOG00000029885 | Stag2 |
| ENSRNOG00000007219 | LOC103692716 |
| ENSRNOG00000050437 | Bod1l1 |
| ENSRNOG00000016152 | Dek |
| ENSRNOG00000054204 | Gria2 |
| ENSRNOG00000059894 | Hmmr |
| ENSRNOG00000005070 | Spopl |
| ENSRNOG00000048577 | Zfp955a |
| ENSRNOG00000017466 | Kif5b |
| ENSRNOG00000004521 | Prpf39 |
| ENSRNOG00000008744 | Cops2 |
| ENSRNOG00000006391 | Smarcad1 |
| ENSRNOG00000004864 | Prpf40a |
| ENSRNOG00000007998 | Ssb |
| ENSRNOG00000003434 | Ro60 |
| ENSRNOG00000060479 | Sp3 |
| ENSRNOG00000013176 | Far1 |
| ENSRNOG00000055631 | Zfp280d |
| ENSRNOG00000009891 | Pcf11 |
| ENSRNOG00000005624 | Zfx |
| ENSRNOG00000021669 | Mybl1 |
| ENSRNOG00000020624 | Acadsb |
| ENSRNOG00000014031 | Esco1 |
| ENSRNOG00000003545 | Uchl5 |
| ENSRNOG00000037227 | Yes1 |
| ENSRNOG00000010737 | Mbnl2 |
| ENSRNOG00000016705 | Prpf4b |
| ENSRNOG00000022502 | Nsrp1 |
| ENSRNOG00000036911 | Bicd1 |
| ENSRNOG00000008658 | Mitf |
| ENSRNOG00000005021 | Orc4 |
| ENSRNOG00000026319 | Akap9 |
| ENSRNOG00000026044 | Prrg1 |
| ENSRNOG00000011137 | Zbtb41 |
| ENSRNOG00000003712 | Ppp4r3b |
| ENSRNOG00000047137 | Erbin |
| ENSRNOG00000022521 | Ddias |
| ENSRNOG00000025604 | Atad2 |
| ENSRNOG00000006967 | Xiap |
| ENSRNOG00000002874 | AABR07065078 |
| ENSRNOG00000000082 | Hltf |
| ENSRNOG00000010999 | Cep295 |
| ENSRNOG00000051756 | Zfp62 |
| ENSRNOG00000014605 | Lig4 |
| ENSRNOG00000056128 | AABR07064719 |
| ENSRNOG00000015255 | Haus3 |
| ENSRNOG00000005123 | Emc2 |
| ENSRNOG00000009340 | Zbtb6 |
| ENSRNOG00000029841 | Cdh19 |
| ENSRNOG00000032735 | Srek1 |
| ENSRNOG00000019875 | Matr3 |
| ENSRNOG00000002835 | Luc7l3 |
| ENSRNOG00000033065 | Rad50 |
| ENSRNOG00000000796 | Ranbp2 |
| ENSRNOG00000000073 | Tmed5 |
| ENSRNOG00000014501 | Zfp638 |
| ENSRNOG00000028137 | Mki67 |
| ENSRNOG00000007673 | Ppig |
| ENSRNOG00000020479 | Pik3c2a |
| ENSRNOG00000004496 | Rock2 |
| ENSRNOG00000031092 | Rock1 |
| ENSRNOG00000011261 | Ttc14 |
| ENSRNOG00000021903 | Atad5 |
| ENSRNOG00000021525 | Nbeal1 |
| ENSRNOG00000008782 | Pnisr |
| ENSRNOG00000030572 | Smc5 |
| ENSRNOG00000002625 | Ptpn4 |
| ENSRNOG00000031216 | AABR07055776 |
| ENSRNOG00000016298 | Lysmd3 |
| ENSRNOG00000008055 | Ccne2 |
| ENSRNOG00000001111 | Brca2 |
| ENSRNOG00000024372 | Cwf19l2 |
| ENSRNOG00000029956 | AABR07033023 |
| ENSRNOG00000056462 | Tmf1 |
| ENSRNOG00000036913 | Resf1 |
| ENSRNOG00000004696 | Arhgap5 |
| ENSRNOG00000051922 | AABR07059663 |
| ENSRNOG00000007202 | Sema3d |
| ENSRNOG00000052444 | Samd9 |
| ENSRNOG00000056907 | Nipbl |
| ENSRNOG00000002827 | Rbfox1 |
| ENSRNOG00000007859 | Cep83 |
| ENSRNOG00000051238 | Mphosph8 |
| ENSRNOG00000002394 | Tpr |
| ENSRNOG00000021681 | Eea1 |
| ENSRNOG00000021776 | Cenpc |
| ENSRNOG00000010155 | Pcm1 |
| ENSRNOG00000015921 | Esco2 |
| ENSRNOG00000052406 | LOC102552527 |
| ENSRNOG00000004908 | Smc6 |
| ENSRNOG00000006487 | Casp8ap2 |
| ENSRNOG00000002280 | Sh3bgrl |
| ENSRNOG00000022325 | Smc2 |
| ENSRNOG00000012067 | Fam111a |
| ENSRNOG00000043151 | Cntln |
| ENSRNOG00000057497 | AABR07042936 |
| ENSRNOG00000024671 | Dmxl1 |
| ENSRNOG00000022845 | Cep70 |
| ENSRNOG00000003787 | Lcorl |
| ENSRNOG00000038572 | Ncapg |
| ENSRNOG00000009208 | Pibf1 |
| ENSRNOG00000007315 | Thoc2 |
| ENSRNOG00000025539 | Vps13a |
| ENSRNOG00000040279 | Slf1 |
| ENSRNOG00000054901 | Rif1 |
| ENSRNOG00000057732 | AABR07043288 |
| ENSRNOG00000019127 | Zfp606 |
| ENSRNOG00000016391 | Arid4b |
| ENSRNOG00000011440 | Ccdc39 |
| ENSRNOG00000004268 | Zfp386 |
| ENSRNOG00000033134 | Mef2c |
| ENSRNOG00000031031 | Zfp292 |
| ENSRNOG00000046420 | AABR07006458 |
| ENSRNOG00000003346 | Fancb |
| ENSRNOG00000057231 | Ddx3 |
| ENSRNOG00000049751 | LOC100912163 |
| ENSRNOG00000004777 | Esf1 |
| ENSRNOG00000047145 | LOC102551340 |
| ENSRNOG00000008652 | Phip |
| ENSRNOG00000056703 | Atrx |
| ENSRNOG00000009503 | Depdc1 |
| ENSRNOG00000001956 | Dzip3 |
| ENSRNOG00000056374 | AC097784 |
| ENSRNOG00000051606 | LOC100360380 |
| ENSRNOG00000042826 | Zfp52 |
| ENSRNOG00000010891 | Lrrcc1 |
| ENSRNOG00000005037 | Kif18a |
| ENSRNOG00000000823 | Gcc2 |
| ENSRNOG00000027035 | Sgo2 |
| ENSRNOG00000004057 | Ccdc88a |
| ENSRNOG00000006833 | Rb1cc1 |
| ENSRNOG00000056036 | LOC103691005 |
| ENSRNOG00000056458 | Cep290 |
| ENSRNOG00000006545 | Septin7 |
| ENSRNOG00000009339 | Cenpe |
| ENSRNOG00000033792 | RGD1565622 |
| ENSRNOG00000003388 | Cenpf |
| ENSRNOG00000018929 | Kif20b |
| ENSRNOG00000054488 | LOC500584 |
| ENSRNOG00000057622 | Rbm41 |
| ENSRNOG00000038960 | RGD1309362 |
| ENSRNOG00000023093 | Mis18bp1 |
| ENSRNOG00000029078 | Hmgn5b |
| ENSRNOG00000051867 | Zfp600 |
| ENSRNOG00000026569 | LOC102555377 |
| ENSRNOG00000010274 | Smc4 |
| ENSRNOG00000054392 | AABR07055826 |
| ENSRNOG00000048864 | LOC100125368 |
| ENSRNOG00000008075 | Ift74 |
| ENSRNOG00000024545 | Ccdc18 |
| ENSRNOG00000038225 | Fbxo15 |
| ENSRNOG00000060100 | Knl1 |
| ENSRNOG00000051570 | AABR07001910 |
| ENSRNOG00000032517 | Cntn6 |
| ENSRNOG00000039674 | AABR07034657 |
| ENSRNOG00000013662 | Erich6 |
| ENSRNOG00000047719 | LOC103689927 |
| ENSRNOG00000059679 | LOC103692165 |

**Table.S7** 49 DEGs between OGD/R + PBS and OGD/R + rrTSG-6 groups.

| gene_id | gene_name |
| --- | --- |
| ENSRNOG00000047719 | LOC103689927 |
| ENSRNOG00000049593 | Wbp11l1 |
| ENSRNOG00000061733 | LOC103690156 |
| ENSRNOG00000047746 | AABR07000398 |
| ENSRNOG00000048489 | AABR07002775 |
| ENSRNOG00000033625 | AABR07027015 |
| ENSRNOG00000060100 | Knl1 |
| ENSRNOG00000006545 | Septin7 |
| ENSRNOG00000010274 | Smc4 |
| ENSRNOG00000018929 | Kif20b |
| ENSRNOG00000006487 | Casp8ap2 |
| ENSRNOG00000003388 | Cenpf |
| ENSRNOG00000009339 | Cenpe |
| ENSRNOG00000004057 | Ccdc88a |
| ENSRNOG00000056703 | Atrx |
| ENSRNOG00000032307 | Dsel |
| ENSRNOG00000036913 | Resf1 |
| ENSRNOG00000050647 | Hspa1b |
| ENSRNOG00000006833 | Rb1cc1 |
| ENSRNOG00000016298 | Lysmd3 |
| ENSRNOG00000051922 | AABR07059663 |
| ENSRNOG00000002609 | Ero1b |
| ENSRNOG00000012067 | Fam111a |
| ENSRNOG00000016677 | Csnk1g3 |
| ENSRNOG00000028243 | Derl3 |
| ENSRNOG00000056462 | Tmf1 |
| ENSRNOG00000021669 | Mybl1 |
| ENSRNOG00000003242 | Gulp1 |
| ENSRNOG00000038933 | Spcs3 |
| ENSRNOG00000013002 | Gpbp1 |
| ENSRNOG00000000397 | Ccar1 |
| ENSRNOG00000013176 | Far1 |
| ENSRNOG00000037462 | Eml6 |
| ENSRNOG00000023356 | Eif5b |
| ENSRNOG00000002618 | Ivns1abp |
| ENSRNOG00000027770 | Trpm3 |
| ENSRNOG00000045771 | Chl1 |
| ENSRNOG00000032303 | LOC108349682 |
| ENSRNOG00000019189 | Acat2 |
| ENSRNOG00000032348 | LOC103690996 |
| ENSRNOG00000039249 | AABR07035539 |
| ENSRNOG00000007081 | Xdh |
| ENSRNOG00000049667 | AABR07030375 |
| ENSRNOG00000059663 | LOC100910755 |
| ENSRNOG00000045654 | LOC108348108 |
| ENSRNOG00000048982 | LOC100912282 |
| ENSRNOG00000045965 | N4bp3 |
| ENSRNOG00000060898 | LOC100910418 |
| ENSRNOG00000050922 | Nupr1l1 |
